# DrugPTM-Bench: A Large-Scale Dataset for Predictive Modeling of Drug-Induced Cell Type-Specific Protein Post-Translational Modifications

**DOI:** 10.3390/info17111079

**Published:** 2026-10-31

**Authors:** Amitesh Badkul, Li Xie, Mohammadsadeq Mottaqi, Lei Xie

**Affiliations:** 1 Ph.D. Program in Computer Science, The Graduate Center, The City University of New York, New York, NY 10016, USA; 2 Department of Computer Science, Hunter College, The City University of New York, New York, NY 10065, USA; 3 Ph.D. Program in Biochemistry, The Graduate Center, The City University of New York, New York, NY 10016, USA; 4 School of Pharmacy and Pharmaceutical Sciences & Center for Drug Discovery, Northeastern University, Boston, MA 02115, USA

**Keywords:** post-translational modifications, deep learning, phosphoproteomics, benchmark dataset, computational drug discovery

## Abstract

Protein post-translational modifications (PTMs), particularly phosphorylation, regulate cellular signaling, drug response, and disease-associated pathway activity. However, existing PTM resources are largely static and do not capture how PTM regulation varies across drug, dose, time, cellular context, and modified site. We present DrugPTM-Bench, a curated benchmark derived from decryptM dose-dependent PTM measurements, standardizing site-level drug response across seven cancer cell lines, 27 drugs, 11,167 proteins, six time points, 16 dosage levels, and pEC50 potency values. Reflecting the central role of phosphorylation in drug-responsive signaling, DrugPTM-Bench is phosphorylation-dominant, with 99.5% phosphorylation events. We formulate a protein-disjoint out-of-distribution classification task to predict whether a PTM site is up-regulated, downr-egulated, or unchanged following drug treatment. Our results show that drug-induced PTM regulation is a highly imbalanced and context-dependent prediction problem: baseline machine learning and deep learning models fail to robustly recover rare regulated events, while standard rebalancing strategies improve recall only at the cost of precision and overall F1-score. Beyond classification, DrugPTM-Bench enables pEC50 regression, mechanism-of-action prediction from PTM fingerprints, and drug-specific PTM-site sensitivity ranking.

## 1. Introduction

Protein post-translational modifications (PTMs) are biochemical processes that regulate protein function, stability, localization, and interactions through chemical alterations. These modifications typically involve the addition of functional groups such as acetyl, methyl, or phosphoryl to specific amino acids [1-3]. PTMs regulate essential biological functions, including cell differentiation, metabolism, and immune response. They also influence signal transduction, gene expression, DNA repair, and the cell cycle. By influencing protein conformation, structure, lifespan, activity, charge, solubility, and interactions with DNA, RNA, and other proteins, PTMs ultimately shape cellular function and phenotypic outcomes [2,4]. These modifications occur at different stages of biological processes across multiple cellular compartments, including the Golgi apparatus, endoplasmic reticulum, nucleus, and cytoplasm [5].

Beyond their fundamental biological roles, PTMs also modulate signaling pathways, create novel druggable sites, and enhance the specificity of therapeutic interventions [6]. Dysregulation of PTMs is implicated in numerous diseases, particularly cancer, neurological disorders, and metabolic syndromes, making them crucial therapeutic targets. For example, phosphorylation dysregulation is a hallmark of cancer, leading to the targeting of kinases in drug development [7]. Inhibiting overactive kinases can prevent the phosphorylation of key proteins involved in uncontrolled cell growth and survival [8]. Similarly, targeting ubiquitination pathways, such as proteasome inhibitors, has led to successful treatments for multiple myeloma [9]. Aberrant PTMs can also contribute to protein misfolding and aggregation, as seen in neurodegenerative disorders like Alzheimer’s and Parkinson’s, where modifying PTM pathways presents a promising therapeutic approach [10]. Drug-induced PTM changes are pharmacologically informative because they provide dynamic readouts of pathway perturbation that can help illuminate target engagement and mechanism of action [11]. Despite their significance, only about 7% of biological systems of PTMs have been explored using small molecule ligands, highlighting the vast, uncharted chemical space for PTM-targeted drug development [6].

Recent advances in artificial intelligence have transformed protein modeling. Protein language models (PLMs) such as ESM-2 [12] and ProtTrans [13] learn rich sequence representations from large-scale UniProt databases, capturing structural, functional, and evolutionary information without requiring explicit structural labels. These representations have been successfully applied to a range of downstream tasks, including structure prediction, function annotation, and variant effect prediction. In parallel, deep learning methods for PTM-site prediction have evolved from convolutional architectures such as MusiteDeep [14] to transformer-based approaches that leverage pretrained protein embeddings [15,16]. On the small-molecule side, molecular encoders such as UniMol [17] provide pretrained 3D-aware drug representations that capture stereochemical and electronic properties. Despite these advances, existing PTM prediction models focus almost exclusively on static site annotation rather than dynamic, context-dependent regulation. Bridging this gap requires both appropriate data resources and benchmark tasks that explicitly model drug-induced PTM responses.

To better characterize PTMs, researchers rely on experimental techniques such as mass spectrometry (MS) coupled with immunoaffinity enrichment, which enables precise identification and quantification of protein modifications [18]. Another widely used approach, Proximity ligation assay (PLA), employs antibody-based proximity detection combined with DNA amplification for signal enhancement [3]. However, both methods have significant drawbacks. MS is expensive and requires extensive sample preparation, while PLA depends on antibody specificity and availability, limiting its applicability [19]. The ability to systematically characterize PTMs is critical for understanding cellular regulation, drug response, and disease pathology. However, experimental PTM profiling remains costly and technically demanding, which has motivated the development of computational approaches for PTM prediction and analysis. In contrast to experimental methods, computational PTM prediction provides a fast and low-cost strategy for proteome-wide annotation and experimental design, and machine learning has become an option for PTM-site prediction [3,20,21]. While machine learning (ML) and deep learning (DL) have significantly improved PTM prediction and analysis [14-16,22], existing PTM databases including dbPTM, PhosphoSitePlus, and BioGRID, primarily capture static PTM annotations rather than dynamic, drug-perturbed PTM responses [23-25]. As a result, they are limited in their ability to support modeling of how PTM states change across drug, dose, time, and cellular context. Recent efforts, such as decryptM [21], have addressed this gap by systematically profiling dose- and time-resolved PTM responses across multiple drugs and cell lines, generating over 1.8 million dose–response curves. Importantly, decryptM shows that drug-regulated PTMs can shed light on target engagement and drug mechanism of action, underscoring the pharmacological relevance of dynamic PTM measurements [21].

While decryptM provides comprehensive, quantitative PTM profiling across drugs, doses, cell lines, and time points, it is not organized as a benchmark for predictive modeling: it lacks standardized tasks, curated splits, and explicit labels for site-level regulatory outcomes required for machine learning evaluation.

To bridge this gap, we introduce DrugPTM-Bench, a curated benchmark that transforms dose-dependent PTM (ddPTM) measurements from the decryptM resource into standardized, site-level prediction tasks enriched with explicit experimental context. DrugPTM-Bench captures PTM regulation across cell lines, drugs, dosage levels, and time points, and also retains pEC50 values, enabling both classification and regression-oriented formulations. In the present work, we focus primarily on PTM regulation classification: given a PTM site, a drug, and its treatment condition, the task is to predict whether the site is up-regulated, down-regulated, or unchanged following treatment. This formulation is pharmacologically meaningful because drug-induced PTM changes are direct readouts of perturbed signaling pathways and therefore relevant to understanding drug response [11,20,21,26].

Another contribution of DrugPTM-Bench is that it exposes a biologically realistic imbalanced-learning regime. Across PTM-chemical pairs, the majority of sites exhibit neutral regulation, creating substantial class imbalance when predicting regulatory outcomes. In some benchmark settings, the neutral-to-minority ratio reaches at least 165:1.

This matters computationally because imbalanced data are known to bias classifiers toward majority classes, while common resampling strategies can introduce noise or degrade decision boundaries. Most work on imbalance in machine learning addresses this problem through resampling or augmentation strategies, ranging from classical methods such as the synthetic minority oversampling technique (SMOTE) [27] and the adaptive synthetic sampling approach (ADASYN) [28] to more recent contrastive-learning-based approaches [29,30]. However, these methods are typically developed and evaluated in domains where the data structure differs substantially from biological perturbation data, and synthetic resampling can be especially problematic when minority examples are sparse and biologically heterogeneous. Existing imbalance benchmarks such as CIFAR-100-LT [31] and ImageNet-LT [32] are also rooted in computer vision and do not capture the context dependence or label structure of PTM regulation data. Consistent with this pattern, the baseline ML and DL models in our benchmark struggle to recover minority regulation classes, and rebalancing improves recall only at the expense of precision and overall F1-score. DrugPTM-Bench therefore serves first as a benchmark for dynamic drug-induced PTM regulation modeling, and second as a benchmark for learning under imbalanced, context-dependent biological data.

In summary, our main contributions are as follows: (1) We introduce DrugPTM-Bench, a large-scale, standardized benchmark dataset for drug-induced, context-dependent PTM regulation, constructed from decryptM-derived ddPTM measurements. (2) We formally define and benchmark the site-level PTM regulation prediction task, providing standardized splits and baseline ML/DL results under extreme class imbalance. (3) We empirically demonstrate and analyze the limitations of existing imbalance-handling methods in this biological context. In addition, the benchmark is explicitly curated to retain pEC50 potency values, drug perturbation profiles, and site-level context, so that the resource supports and motivates three additional predictive tasks enabled by DrugPTM-Bench beyond the primary classification task, including pEC50 potency regression, MoA prediction from PTM perturbation profiles, and drug-specific PTM site sensitivity ranking (see Supplementary Material).

## 2. Results and Discussion

### 2.1. Dataset

#### 2.1.1. Existing Dataset Gap

Existing PTM datasets have primarily evolved along three axes: (1) expansion of curated PTM sites, (2) functional and mechanistic annotation, and (3) integration with biological networks. Early resources, such as Phospho.ELM [33], focused on cataloging phosphorylation sites, providing conservation scores and disorder metrics but lacking drug-disease associations. PhosphoSitePlus [25] expanded PTM annotations to over 450,000 sites, linking them to disease mutations, yet it retained a static view of PTMs without capturing dose- and time-dependent drug effects. EPSD 2.0 [34] integrated functional annotations for 88,074 phosphorylation events, incorporating multi-omics data, yet its drug-PTM associations were inferred rather than experimentally validated. dbPTM [23] further integrated 2.8 million PTM sites with kinase-substrate and E3 ligase interactions, allowing hypothesis generation about upstream regulators, but without providing explicit drug perturbation effects over time. Similarly, ActiveDriverDB [35] maps disease-associated mutations to PTM regions but lacks information on how pharmacological interventions modulate PTMs. As summarized in Table 1, no existing dataset systematically captures dose- and time-resolved drug-induced PTM regulations, highlighting the need for DrugPTM-Bench as a dedicated resource for studying dynamic PTM perturbations in response to drug treatments.

#### 2.1.2. DrugPTM-Bench Curation and Benchmark Construction

DrugPTM-Bench was constructed from processed dose-dependent PTM (ddPTM) measurements derived from decryptM [21]. Experimental sample preparation, LC-MS/MS acquisition, peptide identification, protein inference, and quantitative processing were performed upstream in decryptM using MaxQuant. DrugPTM-Bench therefore begins from the processed measurement export and applies benchmark-specific filtering, sequence mapping, label construction, and standardized evaluation partitioning. An overview of this workflow is shown in Figure 1. The original decryptm source contains 10,733,521 PTM measurement records together with PTM identity, peptide sequence, protein annotations, cell line, drug, dosage, treatment time, signal ratio (SR), and associated dose–response information. During benchmark-specific quality control, records with missing or non-numeric SR values were removed, followed by records with SR = 0, for which log_2_(SR) is undefined. This filtering retained 10,555,440 measurements with valid signal ratios. The processed quantitative measurements inherited from decryptM were retained, and SR values were subsequently analyzed on the log_2_ scale for regulation-label assignment. To construct sequence-resolved benchmark instances, PTM-containing peptides were mapped to full-length protein sequences using the protein assignments reported by MaxQuant [37]. Because peptides generated by bottom-up proteomics may be shared across multiple proteins, protein assignment can be ambiguous. DrugPTM-Bench therefore uses a leading-protein-first mapping strategy: each PTM-containing peptide is first mapped to the MaxQuant leading-protein assignment, and when a valid sequence match cannot be obtained, additional candidate proteins reported by MaxQuant are examined. The peptide-local modification position is then converted to the corresponding PTM coordinate on the full-length protein sequence. Entries for which an appropriate protein sequence could not be recovered, including unavailable or deleted identifiers from ENSEMBL [38], were excluded. Applying these steps to the protein annotations yields the following retained counts. Of the 11,167 unique leading-protein accessions present in the source export, 305 were removed because their only associated measurements carried missing or non-numeric signal ratios, leaving 10,862. A further 14 accessions occur exclusively within multi-accession (ambiguous) leading-protein strings and were therefore not retained as standalone proteins, leaving 10,848. Of these, 21 could not be mapped because their identifiers were no longer available in ENSEMBL [38]. The curated benchmark therefore retains 10,827 leading proteins with explicit PTM-site coordinates, drawn from 11,167 source accessions, alongside the 10,555,440 retained measurement records described above. Throughout this paper, 11,167 proteins and 10,733,521 records denote source counts prior to quality control, whereas 10,827 proteins and 10,555,440 records denote the counts retained in the curated benchmark.

This procedure provides explicit protein-level PTM-site coordinates required for sequence-based feature construction while retaining the original MaxQuant-based protein inference. To quantify protein-mapping ambiguity, we examined the candidate protein assignments associated with each measurement record. Approximately 20.6% of records contained a single candidate protein, 21.1% contained multiple candidates corresponding to the same base protein accession, and 58.3% contained candidates spanning multiple distinct protein accessions. These values quantify ambiguity in the source protein-inference annotations rather than mapping-error rates, because DrugPTM-Bench uses the MaxQuant leading-protein assignment as the primary mapping target. Following curation and sequence mapping, quantitative PTM responses were converted into the three benchmark regulation classes using the operational rule log_2_ (SR) > 1 for *Up*, log_2_(SR) < −1 for *Down*, and −1 ≤ log_2_ (SR) ≤ 1 for *Neutral*. The rationale and sensitivity of this labeling rule are described separately in Section 2.1.4 and Supplementary Section Threshold Sensitivity Analysis. Finally, the curated benchmark was organized into protein-disjoint training, validation, and test partitions using a 70:20:10 split, ensuring that no protein identity occurs in more than one partition. The motivation and evaluation implications of this split are described in Section 2.2.

#### 2.1.3. Dataset Overview

DrugPTM-Bench is constructed from decryptM-derived ddPTM (dose-dependent posttranslational modification) measurements and comprises 10,733,521 PTM records across seven human cancer cell lines, 27 unique drugs, 11,167 proteins, six treatment time points, and 15 non-zero dosage levels. The cell-line panel includes A431 (epidermoid carcinoma), A549 (lung adenocarcinoma), BT-474 and MDA-MB-175 (breast carcinoma), HeLa (cervical adenocarcinoma), K562 (chronic myeloid leukemia), and RPMI8226 (multiple myeloma). In addition to PTM identities and protein sequence context, the benchmark retains the key experimental variables required for predictive modeling, including cell line, drug, dosage, treatment time, signal ratio, and negative logarithmic half-maximal effective concentration (pEC50) values. Figure 2 provides a high-level overview of the benchmark composition and response distributions.

As shown in Figure 2A, DrugPTM-Bench is dominated by phosphorylation measurements (99.5%), while acetylation accounts for only a small minority of records (0.5%). Given this strong skew in PTM type composition, the current benchmark experiments focus primarily on phosphorylation-based regulation modeling. Phosphorylation is one of the most prevalent and extensively characterized PTMs and is a central mediator of drug-responsive signaling networks. Nevertheless, the present benchmark should be interpreted primarily as a phosphoproteomics benchmark rather than as an equally comprehensive resource for all PTM classes [39]. This unevenness reflects the underlying experimental design of the source perturbation proteomics data rather than any additional balancing imposed during benchmark construction. The strong dominance of phosphorylation has important implications for the benchmark’s applicability to other PTM types. Phosphorylation is primarily regulated by kinases and phosphatases, and its regulatory logic. Consequently, models trained predominantly on phosphorylation data may learn phosphorylation-specific regulatory patterns that do not readily generalize to other PTM types. DrugPTM-Bench should therefore be interpreted primarily as a phosphoproteomics benchmark in its current form. Nevertheless, the benchmark framework itself, including the data schema, labeling procedure, evaluation protocol, and model architecture, is PTM-type-agnostic and can be extended to additional modification types as comparable dose- and time-resolved perturbation data becomes available.

The benchmark captures PTM responses over six post-treatment time points and 15 non-zero drug concentrations, together with untreated control measurements. As shown in Figure 2C, measurements are concentrated at earlier response windows, particularly 30 and 60 min, indicating that the dataset is enriched for acute drug-induced PTM responses.

Figure 2D shows that dosage coverage is broad, ranging from 0.01 to 100,000 μg/mL, with the highest recorded density concentrated in the intermediate dosage range. Together, these two additional dimensions make DrugPTM-Bench suitable for studying how PTM regulation varies across both treatment duration and drug exposure level. To quantify PTM response magnitude, DrugPTM-Bench uses the signal ratio between treated and untreated conditions, which is subsequently analyzed on the log_2_ scale. Figure 2E shows that the log_2_(signal ratio) distribution is sharply centered near zero, indicating that most PTM sites remain close to unchanged under a given treatment condition, while a smaller subset exhibits strong positive or negative shifts. Finally, DrugPTM-Bench retains pEC50 values as a measure of drug potency at the PTM-site level. As shown in Figure 2F, pEC50 values span a broad range across cell lines, reflecting substantial variation in the strength and sensitivity of PTM responses under drug perturbation.

#### 2.1.4. Signal Ratio, Regulation Labels, and Benchmark Tasks

To quantify drug-induced PTM response, DrugPTM-Bench uses the signal ratio (SR), defined as the ratio of PTM signal intensity in the drug-treated condition to that in the untreated control condition:

(1) $$\text{Signal Ratio}=\frac{\text{Signal Intensity with Drug}}{\text{Signal Intensity without Drug}}$$

This quantity provides a direct measure of the magnitude and direction of drug-induced PTM change at a site. Because PTM abundances span a wide dynamic range, we analyze SR on the log_2_ scale. The log_2_ transformation is standard in proteomics because it compresses large fold-change ranges and renders up- and down-regulation symmetric around zero, thereby making the response more suitable for statistical analysis and machine learning.

Figure 2E shows that the log_2_(SR) distribution is sharply centered near zero, with most PTM sites exhibiting little or no response under a given treatment condition. A closer examination of the interval between −3 and 3 shows that 136,925 PTM-drug pairs (1.2% of all pairs) have log_2_ (SR) > 1, whereas 168,538 pairs (1.5%) have log_2_ (SR) < −1. Following established quantitative phosphoproteomics practice, we use ∣ log_2_ (SR)∣ > 1 corresponding to a greater than two-fold increase or decrease relative to untreated control, as a magnitude-based threshold for defining regulated PTM responses [40-42]. Two-fold response criteria have been used extensively in quantitative phosphoproteomic studies to identify strongly modulated phosphorylation events, with prior work also demonstrating that this cutoff can exceed typical measurement variability and provide a conservative separation from background noise. Accordingly, each PTM measurement is assigned one of three regulation labels: *Up* for log_2_ (SR) > 1, *Down* for log_2_ (SR) < −1, and *Neutral* for −1 ≤ log_2_ (SR) ≤ 1. This converts the quantitative PTM response into an explicit three-class prediction problem.

#### 2.1.5. Class Imbalance Landscape

A central property of DrugPTM-Bench is the severe class imbalance inherent to drug-induced PTM regulation. As shown in Figure 3B, the vast majority of PTM measurements remain neutral across all cell lines, with only a small fraction of sites classified as up- or down-regulated. However, this imbalance is not uniform across cellular contexts. In the aggregate view across all dosage conditions, A549 exhibits the strongest overall imbalance, with only 1.1% of measurements falling into the regulated classes, whereas RPMI8226 shows the weakest overall imbalance, with 8.3% regulated sites. K562 is also strongly imbalanced in the aggregate view, with only 1.5% regulated measurements. Figure 3A further shows that imbalance depends strongly on dosage. The most extreme ratios occur at lower dosage bins, where regulated PTM events are especially sparse. For example, cell line K562 reaches a neutral-to-regulated ratio of 165:1 in the lowest dosage bin, and A549 reaches 131:1 in the 0.01–0.1 μg/mL range. As dosage increases, the imbalance generally decreases across most cell lines, indicating that stronger drug exposure perturbs a larger fraction of PTM sites. This pattern is consistent with dose-resolved phosphoproteomic studies showing that increasing drug concentration enhances target and pathway engagement and propagates perturbations more broadly through signaling networks [21]. Nevertheless, even at the highest dosage bins, the neutral class remains dominant in nearly all cell lines. For the purposes of benchmarking, the key implication is that DrugPTM-Bench does not present a single fixed imbalance ratio, but instead spans a spectrum of imbalance conditions across cell lines and dosage levels. Treatment duration can also alter the breadth of detectable PTM regulation because early responses may reflect direct target-proximal signaling events, whereas later time points can capture secondary pathway propagation, feedback regulation, and adaptive cellular response [21,43].

#### 2.1.6. Drug-Induced Perturbation Landscape

Figure 4 summarizes how strongly different drugs perturb the PTM landscape across cellular contexts. Figure 4A aggregates all non-neutral PTM responses, defined by the benchmark regulation labels (*Up* and *Down*), for each drug–cell line combination. Bubble size represents the total number of regulated PTM sites, whereas bubble colour reflects the balance between up- and down-regulation, allowing perturbation magnitude and directionality to be visualized simultaneously. This view shows that drug-induced PTM perturbation is highly heterogeneous across cellular contexts: some drug–cell line combinations induce broad PTM reprogramming, whereas others affect only a comparatively small subset of sites. Such heterogeneity with the dose-resolved PTM measurements can reveal target engagement, pathway engagement, and mechanism-of-action-related signatures in a context-dependent manner.

Figure 4B ranks the most perturbing drugs in the benchmark. The highest-ranking compounds are enriched for drugs with broad effects on chromatin regulation, proteostasis, and signalling, including the CBP / p300 histone acetyltransferase inhibitor A-485 [44], the proteasome inhibitors bortezomib and carfilzomib [45], the multi-target HDAC/EGFR/HER2 inhibitor CUDC-101 [46], the HDAC inhibitors vorinostat and romidepsin [47,48], the pan-kinase inhibitor staurosporine [49], and the HER2-targeted monoclonal antibody trastuzumab [50]. A-486 should not be described as an active HAT inhibitor [44]; rather, it is better treated as the inactive analog or control compound associated with A-485. This spectrum of mechanisms suggests that DrugPTM-Bench contains a wide range of perturbational regimes, from chromatin-directed and proteostasis-directed responses to receptor- and kinase-driven signaling effects, thereby increasing the benchmark’s value for modeling context-dependent PTM regulation.

#### 2.1.7. Biological Relevance of DrugPTM-Bench

Beyond the global perturbation patterns described above, we examined the site-level regulatory classes/states defined in DrugPTM-Bench and how these preserve established drug-response biology.

A representative example is provided by BCR-ABL signaling in K562 cells. K562 is a BCR-ABL-positive chronic myeloid leukemia model, and inhibition of BCR-ABL is known to suppress phosphorylation of the kinase itself and several downstream signaling components. When considering the drug dasatinib for the K562 cell line, the MAPK1/ERK2 phosphopeptide containing Y187 is predominantly Neutral at the lower sampled doses but becomes consistently Down at higher exposure levels. Similar dose-dependent transitions are observed for the SHC1 Y427 and the BCR Y177 sites. These patterns are consistent with independent quantitative phosphoproteomic analysis of dasatinib-treated K562 cells, which demonstrated suppression of BCR-ABL-associated signaling, including phosphorylation of ABL1, BCR, MAPK1/ERK2, MAPK3/ERK1, and other downstream pathway components [42].

Another such example is illustrated by bortezomib treatment of the multiple myeloma cell line RPMI8226. Proteasome inhibition is known to induce substantial proteotoxic stress in multiple myeloma cells, activating both the unfolded protein response and heat-shock signaling. In RPMI8226 specifically, bortezomib has been shown to induce a heat-shock response and time-dependent HSF1 phosphorylation, while activation of the PERK-eIF2*α*-ATF4 stress axis has also been observed following bortezomib treatment [51,52]. Consistent with this stress-response biology, DrugPTM-Bench contains dose- and time-dependent regulatory transitions in stress-associated phosphoproteins. For example, the HSPB1 phosphopeptide remains Neutral across the early 1–4 h measurements but becomes Up-regulated at higher doses after 8 h and is strongly Up-regulated at 16 h. Similarly, an ATF4 phosphopeptide is Neutral at the lower sampled doses at 8 h but transitions to Up regulation with increasing bortezomib exposure. These observations are consistent at the pathway level with the established proteotoxic-stress response to proteasome inhibition in multiple myeloma [51,53].

Together, these examples illustrate that the categorical formulation used in DrugPTM-Bench preserves known pharmacologically relevant signaling behavior across distinct drugs and cellular backgrounds. Importantly, the resulting Neutral-to-Up or Neutral-to-Down transitions should not be interpreted as exact pharmacological onset concentrations, because they depend on the discrete experimental sampling and the operational ∣ log_2_ (SR)∣ > 1 threshold. Rather, they provide an interpretable representation of context-specific drug response that can be used to evaluate whether predictive models can identify which PTM sites are likely to become regulated under a given perturbational condition. Such predictions could support prioritization of drug-PTM pairs and treatment conditions for follow-up mechanistic studies.

#### 2.1.8. Additional Predictive Tasks Enabled by DrugPTM-Bench

The perturbation landscape described above illustrates that DrugPTM-Bench captures rich, multi-dimensional drug-PTM relationships that extend well beyond the primary classification task. The four task formulations enabled by the benchmark are summarized in Supplementary Material. Specifically, the retention of pEC50 values enables a drug potency regression task at PTM-site resolution, the availability of full drug perturbation profiles across sites enables mechanism-of-action prediction, and the site-level signal ratios per drug-protein pair enable a drug-specific PTM site sensitivity ranking task. Each of these formulations addresses a distinct open problem in computational drug discovery for which no existing benchmark currently operates at PTM-site resolution. A structured description of each task, including its input and output specification, biological motivation, gap relative to existing benchmarks, and key supporting literature is provided in Supplementary material, specifically in Table S1.

### 2.2. Benchmark

#### 2.2.1. Problem Description

Given a PTM site and a drug, the objective is to predict the regulatory outcome, whether the PTM site is up-regulated, down-regulated, or remains neutral following treatment. The cell line, dosage, and treatment timepoint serve as stratifiation variables as opposed to model inputs. Each unique combination of cell line, dosage, and timepoint constitutes a separate subproblem with its own class distribution and imbalance regime. This design allows us to study how prediction difficulty varies across biological contexts while keeping the model inputs fixed. To illustrate the impact of DrugPTM-Bench’s class imbalance which ranges from low to medium to extreme, we frame this as a classification task, where models must learn to distinguish between rare regulatory events and dominant neutral cases. We evaluate the baseline models across three benchmark settings that span a spectrum of class imbalance: Severe (K562 cell line, 0.1 μg/mL, neutral-to-minority ratio ≈ 100:1), Medium (A431 cell line, 10 μg/mL, ≈60:1), and Low (RPMI8226 cell line, 100 μg/mL, ≈5:1). The class counts for each setting are summarized in Table S2 (in the Supplementary Material). To ensure an out-of-distribution (OOD) evaluation, a protein-disjoint split is applied: proteins (groups of PTM sites/rows belonging to the same protein) are randomly partitioned into training, validation, and test sets in a 7:2:1 ratio, guaranteeing that no protein appears in more than one split. This design mimics the real-world scenario in which models must be generalized to previously unseen protein targets. In contrast, a conventional random split may place measurements from the same protein in both training and test sets, allowing models to benefit from protein-specific knowledge from training and likely yielding an overconfident model performance. The protein-disjoint strategy therefore provides a stricter assessment of whether learned representations transfer across proteins rather than primarily exploiting protein identity. As a result, it more directly reflects the intended generalization challenge of DrugPTM-Bench. The split is generated using a fixed random seed of 42. The models’ architecture and hyperparameters are detailed in the Supplement Tables.

We also provide a comprehensive assessment, beyond the three representative imbalance regions, of the models on the 91 unique configurations across the multiple cell lines, timepoints and dosages.

#### 2.2.2. Feature Representation

Protein PTM site representations are derived from ESM2 [12], a protein language model (PLM) that encodes residue-level sequence context. Chemical representations are obtained from UniMol [17], a pretrained molecular encoder that captures three-dimensional molecular geometry. For the machine learning (ML) models, the ESM2 and UniMol embeddings are concatenated into a single feature vector. For the deep learning (DL) model, the two modalities are processed by separate encoder branches each consisting of a series of multi-layer perceptron (MLP) layers and fused at a later stage for the final classification.

#### 2.2.3. Models

We evaluate six model configurations spanning two families. In the ML family, we train a decision tree (DT) and a random forest (RF) on the full, unbalanced training set. We additionally train ensemble variants (DT-ensemble, RF-ensemble) in which the neutral class is partitioned into non-overlapping subsets, each combined with all minority-class examples to form a balanced training subset. In this setting, a separate model is trained on each subset and predictions are aggregated by majority vote. In the DL family, we train a dual-encoder model with standard cross-entropy loss (Dual Encoder) and with focal loss [54] (Dual Encoder Focal), which down-weights well-classified majority examples to focus learning on the minority classes. As an additional trivial baseline, we evaluate is the majority-class baseline that always predicts Neutral. By construction, this baseline yields MCC = 0 and Macro F1 ≈ 0.33 we use it as a floor against which the six trained models are compared.

#### 2.2.4. Software and Hardware

All experiments were implemented in Python 3.10 using PyTorch (version 2.1) for deep learning models and scikit-learn (version 1.6) for machine learning models. All model training and inference were performed on a single NVIDIA L40S GPU with CUDA 12.2. A fixed random seed of 42 was used for all experiments to ensure reproducibility.

#### 2.2.5. Evaluation Metrics

The primary evaluation metrics are Macro F1 and Matthews Correlation Coefficient (MCC), which together capture both per-class balance and overall predictive quality under imbalance. Supporting metrics include per-class recall, precision, and area under the precision-recall curve (AUPRC) for the Up and Down classes, which are reported to characterize minority-class detection independently. Accuracy is not reported, as it is dominated by the neutral majority class and provides no meaningful signal in this imbalanced setting. The choice of MCC and Macro F1 over accuracy is motivated by the severe class imbalance in DrugPTM-Bench. MCC accounts for all four quadrants of the confusion matrix (true positives, true negatives, false positives, false negatives) and remains informative even when class distributions are highly skewed, a value of 0 indicates random performance and 1 indicates perfect prediction. Macro F1 assigns equal weight to each class regardless of frequency, preventing the majority class from dominating the score. However, Macro F1 can mask poor minority-class performance when the Neutral class is predicted well, which is why we additionally report per-class precision, recall, and AUPRC. To assess whether models produce useful ranking signal, we additionally report precision-recall curves for the Up and Down classes and prevalence-relative AUPRC, defined as AUPRC divided by the class prevalence in the test set. A value greater than 1 indicates that the model ranks true positives above what would be expected from random ordering. We further quantify ranking quality using top-k enrichment factors: for each model, we rank test-set instances by predicted probability for the Up (or Down) class and compute the ratio of true-positive density in the top k% to the class prevalence.

For each class c∈ {Neutral, Up, Down}, precision and recall are defined as

(2) $${\text{Precision}}_{c}=\frac{{\text{TP}}_{c}}{{\text{TP}}_{c}+{\text{FP}}_{c}},$$

(3) $${\text{Recall}}_{c}=\frac{{\text{TP}}_{c}}{{\text{TP}}_{c}+{\text{FN}}_{c}},$$

where ${\text{TP}}_{c}$, ${\text{FP}}_{c}$, and ${\text{FN}}_{c}$ denote the true-positive, false-positive, and false-negative counts for class c, respectively. The class-specific F1 score is defined as

(4) $$F1_{c}=\frac{2\cdot {\text{Precision}}_{c}\cdot {\text{Recall}}_{c}}{{\text{Precision}}_{c}+{\text{Recall}}_{c}}.$$

Because DrugPTM-Bench is highly imbalanced, we report Macro F1, which assigns equal weight to each class regardless of its frequency:

(5) $$\text{Macro F}1=\frac{1}{|𝒞|}\sum_{c\in 𝒞}F1_{c},$$

where 𝒞 = {Neutral, Up, Down} and ∣𝒞∣ = 3.

For top-k enrichment analysis, the enrichment factor at k% for class c is defined as

(6) $$\text{EF}@k_{c}=\frac{\text{Precision}@k_{c}}{{\text{Prevalence}}_{c}}=\frac{{\text{TP}}_{k,c}∕k}{n_{c}∕N},$$

where ${\text{TP}}_{k,c}$ is the number of true class-c instances among the top-k% of test instances ranked by predicted probability for class c, and $n_{c}∕N$ is the prevalence of class c in the test set.

### 2.3. Evaluation of Machine Learning and Deep Learning Models

#### 2.3.1. Class Imbalance Strongly Constrains Minority-Event Recovery Across Biological Contexts

As shown in Figure 5, across all three settings, the spread in Macro F1 among the six models within a single regime is at most 0.08 (Medium), whereas the shift in MCC between the Severe and Low regimes, holding the model family constant, is far larger in relative terms. In the Severe setting (K562, 0.1 μg, ~100:1 ratio), all six models produce MCC values between −0.009 and 0.013 (Figure 5, bottom panel), a range that is indistinguishable from random. In the Low setting (RPMI8226, 100 μg, ~5:1 ratio), MCC varies from −0.021–0.062 (Figure 5, bottom panel) and minority-class recall reaches up to 0.43 (Table 2). Thus, changing the model architecture within a fixed configuration does not eliminate the substantial differences in difficulty observed across configurations. The observed neutral-to-regulated ratio (or the imabalance ratio) is a property of the benchmark configuration as a result of the underlying biological response distribution, experimental measurement, and the log_2_(SR) threshold used to define regulation classes. Our controlled analyses (Supplementary Section Threshold Sensitivity Analysis) confirm that dosage, treatment duration, and cellular background can substantially alter this ratio. This pattern indicates that the benchmark difficulty is primarily determined by the underlying biology, that is how many PTM sites are genuinely regulated at a given dose, rather than choice of classifier. Explicit rebalancing substantially increases recovery of regulated events for the tree-based models, whereas the standard random forest frequently collapses toward prediction of the dominant Neutral class. Because these comparisons retain the same biological configuration while changing how class imbalance is handled, they provide direct evidence that imbalance is an important source of learning difficulty in DrugPTM-Bench. Reducing the effective imbalance ratio, whether through higher drug doses, longer treatment windows, or targeted enrichment of regulated sites, is therefore one avenue for improving minority-event recovery, alongside algorithmic approaches that address imbalance directly [55].

#### 2.3.2. Models Retain Ranking Signal Even When Hard Classification Is Near-Random

The majority-class baseline (always predicting Neutral) yields MCC = 0 and Macro F1 of 0.332, 0.330, and 0.324 in the Severe, Medium, and Low settings, respectively. RF matches this baseline almost exactly in all three settings (Macro F1 within 0.0005 and MCC = 0), confirming that it collapses to the trivial Neutral-only predictor, while the remaining five models exceed it only modestly in Macro F1 (Table 2).

Despite this near-random hard-classification performance, models retain useful ranking signal. Precision-recall curves for the Up and Down classes (Figure 6) show that several models rise above the class-prevalence baseline at low recall. In the Severe setting, the Dual Encoder achieves a prevalence-relative AUPRC (AUPRC divided by class prevalence) of 7.40 (95% CI 0.72–40.26) for the Up class despite an MCC of only 0.013 (95% CI −0.013–0.064), indicating that its probability scores carry substantial ranking information that is lost when thresholded into hard predictions. Top-k enrichment analysis strengthens this pattern: in the Severe setting, the Dual Encoder achieves an enrichment factor of 11.83× ((95% CI 0.00–30.61)) at the top 1% for the Up class, and RF-ensemble achieves 5.92× ((95% CI 0.00–24.84)) (as seen in Supplementary Figure S2). Confidence intervals throughout are protein-clustered bootstrap intervals (1500 resamples of test-set proteins with replacement), which account for the fact that PTM sites measured on the same protein are not independent observations. (Full results for all three settings and all six models are given in Supplementary Tables S5 and S6.) Both decay rapidly at larger k, indicating that the ranking signal is concentrated in the highest-confidence predictions. In a screening scenario where only the top 1% of predictions can be experimentally validated, the Dual Encoder would identify nearly 12× more true Up-regulated sites than random selection, suggesting that baseline models may be more useful for prioritization than for hard classification.

#### 2.3.3. Rebalancing and Focal Loss Trade Precision for Recall in Opposite Ways, and Neither Dominates

Ensemble rebalancing (DT-ensemble, RF-ensemble) and focal loss (Dual Encoder Focal) both shift the precision–recall trade-off toward higher recall, but through different mechanisms and with different failure modes. As shown in Table 2, in the Severe setting, DT-ensemble raises Recall-Up from 0.000 to 0.235 but collapses Precision-Up to 0.005 (199 of 200 predicted Up events are false positives), while Dual Encoder Focal achieves zero recall on both minority classes and a negative MCC (−0.013). In the Low setting, the picture reverses: Dual Encoder Focal achieves the best MCC (0.062) and the highest combined recall (Recall-Up 0.431, Recall-Down 0.337), while RF-ensemble achieves the highest Macro F1 (0.334) but with Recall-Down of only 0.059. The absence of a single method that dominates across both metrics and all regimes is itself a key finding: it implies that the choice of intervention should be guided by the downstream use case, high-recall screening versus high-precision target prioritization, rather than by a single aggregate score [56].

#### 2.3.4. The Dual Encoder Detects Up-Regulated Sites More Reliably than Down-Regulated Ones, Except Under Focal Loss

Averaged across models, Recall-Up exceeds Recall-Down in all three settings (Severe: 0.088 vs. 0.071; Medium: 0.147 vs. 0.108; Low: 0.298 vs. 0.175). This asymmetry is consistent with the biology of drug-induced phosphorylation: kinase activation events tend to produce large, reproducible fold-changes that are easier to distinguish from background noise, whereas phosphorylation loss can reflect kinase inhibition, phosphatase activation, or indirect network rewiring through distinct and more heterogeneous mechanisms [57]. Focal loss partially reverses this asymmetry: in the Medium setting, Dual Encoder Focal achieves Recall-Down of 0.376 versus Recall-Up of 0.093, suggesting that the loss modulation preferentially rescues the Down class in this regime. Understanding why focal loss selectively amplifies Down-class detection, and whether this reflects a feature-space property of dephosphorylation sites, is an open question for future work.

#### 2.3.5. A Degenerate High-Precision Solution Emerges in the Low Setting, Revealing a Feature-Space Artifact

The standard RF in the Low setting achieves Precision-Up of 1.000 with Recall-Up of 0.001, meaning it predicts exactly one Up event in the entire test set and that prediction happens to be correct. This is not a useful classifier, but it is informative: it suggests that a small subset of Up-regulated sites has a feature signature that is highly separable from the Neutral class, while the vast majority of regulated sites are not. This pattern is consistent with the known structure of drug-induced PTM networks, where a small number of direct substrate sites (e.g., activation loop phosphorylations on the primary drug target) produce strong, reproducible signals, while the broader network of indirect regulation events is far more variable [58]. Future models that explicitly distinguish direct from indirect regulation, for example by incorporating kinase–substrate network priors, may be able to exploit this separability more broadly.

#### 2.3.6. No Single Model Dominates Across the Entire Benchmark

DrugPTM-Bench spans across six cell lines (A431, A549, HeLa, K562, MDA-MB-175, RPMI8226), six timepoints (30–960 min), and 15 dosage levels (0.01–100,000 μg/mL), resulting in 91 unique configurations. Across these 91 unique configurations, we ran our six baseline models. For large-scale evaluation, we consider MCC as the primary metric since, in case of extreme imbalance, it considers all four quadrants of a confusion matrix and hence is useful for comparing the discriminative ability of these models and remains informative under extreme data imbalance. Additionally, we also look at the Minority-class F1 (mean of Up F1 and Down F1) and individual Up/Down F1 scores assess recovery of regulated PTM sites. For any given configuration, we count a particular model to be the best at that configuration if that model has the highest MCC among the rest. Table 3 displays clearly that the best-performing model is the random forest-ensemble model with 30 best runs; however, the Dual Encoder model is close behind it with 22 runs, and the gap between the top two is modest. So the distribution of best performance is not concentrated at one model type rather is spread among the six.

Figure 7 presents the full MCC distribution for each model within every cell line. Several patterns are evident. First, MCC values are low across all models and configurations (range: −0.13 to 0.16, 25% of values are negative), confirming that three-class PTM regulation prediction under protein-disjoint out-of-distribution evaluation is a genuinely difficult task. The win distribution is not uniform across cell lines but instead tracks the underlying imbalance regime. RF-ensemble dominates in the HeLa cell line (13 of 25 configurations) and RPMI8226 (9 of 25), the two cell lines with the lowest mean imbalance ratios (30.9:1 and 30.7:1, respectively) and the most extensive timepoint coverage. In contrast, the Dual Encoder with cross-entropy wins more frequently in A549 (6 of 9) and K562 (6 of 13), where the mean imbalance ratio is substantially higher (168.7:1 and 99.0:1) and only a single timepoint is available. This pattern suggests that ensemble rebalancing is most advantageous when minority classes have sufficient representation for the resampled training subsets to capture meaningful decision boundaries, whereas the Dual Encoder ’s learned feature fusion is more robust when minority examples are extremely sparse. A direct comparison by imbalance regime confirms this: in the medium-imbalance regime (10–50:1), RF-ensemble more than doubles the Dual Encoder’s mean MCC (0.035 vs. 0.015), but in the severe-imbalance regime (>50:1) the relationship reverses, with the Dual Encoder achieving roughly twice the mean MCC of RF-ensemble (0.034 vs. 0.017). In the low-imbalance regime (≤10:1), the two models converge (0.070 vs. 0.062). The advantage of rebalancing/ensembling is therefore imbalance ratio-dependent rather than universal. A striking finding is the behavior of RF: in 86 of 91 configurations (95%), the model collapses to predicting only the Neutral class, producing no Up or Down predictions at all, and yielding MCC of 0. The five configurations where it does not collapse all occur at the lowest imbalance ratios where the minority class is sufficiently represented. This is most likely a direct consequence of majority voting under extreme imbalance: individual trees do occasionally predict the minority classes. However, for a RF to predict minority class, most of its trees should independently agree on the same class, which almost never occurs when the minority signal is sparse. The majority vote thus acts as a low-pass filter that suppresses rare-class predictions. This illustrates why naive ensembling without explicit imbalance handling is insufficient for PTM regulation prediction, and motivates the rebalancing and focal-loss interventions evaluated here. Finally, these results suggest that model choice should depend on the imbalance regime and the intended downstream use, rather than on a single aggregate ranking. In moderate-imbalance settings, where Up and Down classes are sufficiently represented, RF-ensemble provides the strongest overall discrimination. In more severe settings, where regulated sites are extremely sparse, the Dual Encoder appears more suitable. When the goal is exploratory screening and recovering as many regulated sites as possible is more important than maintaining high precision, focal loss and rebalancing provide useful ways to shift the precision-recall trade-off. All this highlights an important feature of the benchmark: PTM regulation prediction does not have one consistent model, but instead varies across a range of imbalance conditions and biological response regions.

## 3. Limitations

Several limitations of DrugPTM-Bench should be considered when interpreting the present results. First, the benchmark is heavily dominated by phosphorylation events (99.5%), limiting the extent to which conclusions from the current experiments can be generalized to other PTM classes such as acetylation, ubiquitination, and methylation. Second, the protein-disjoint split prevents the same protein from appearing across training, validation, and test sets, and it does enforce a low sequence similarity between proteins in different splits (under 3%), however, not completely zero. Moreover, because drugs and experimental contexts are not independently held out, the present evaluation should be interpreted specifically as generalization to unseen protein identities rather than as a comprehensive out-of-distribution evaluation across drugs. Thirdly, two distinct sources of uncertainty affect these results and are handled differently here. Evaluation (sampling) uncertainty, which reflects how much a reported metric would move had a different set of proteins been held out, is quantified for the three representative settings by protein-clustered bootstrap confidence intervals reported in Supplementary Tables S5 and S6. Training variability, which reflects the sensitivity of each fitted model to random initialization and data ordering, is not quantified: because the benchmark comprises 91 configurations, repeating training across multiple seeds for every configuration was outside the computational scope of this study and is left to future work. Fourthly, the empirical evaluation in the present study is intentionally focused on the three-class PTM-regulation task and on reference machine- and deep-learning baselines using pretrained protein and molecular representations. The reported results therefore establish baseline difficulty rather than an upper bound on achievable performance. Lastly, the additional pEC50-regression, mechanism-of-action, and PTM-sensitivity-ranking tasks defined by DrugPTM-Bench remain to be empirically benchmarked.

## 4. Future Work

DrugPTM-Bench opens several directions for future research. First, the additional predictive tasks outlined in Supplementary Table S1 (including pEC50 potency regression, mechanism-of-action prediction from PTM perturbation profiles, and drug-specific PTM-site sensitivity ranking) represent natural extensions of the present classification benchmark. Second, richer protein representations may improve the ability of models to distinguish regulated from neutral PTM sites. Using supervised fine-tuning of protein language models (PLMs) can improve performance of classifiers [59]. PTM-specific studies have likewise demonstrated the benefit of combining pretrained protein representations with local sequence context [60], while recent graph-based approaches have integrated PLM-derived features with protein structural contact information for phosphorylation-site prediction [61]. Future work could therefore investigate residue-level representations, and structure-aware encoders. Third, a complementary direction is the explicit incorporation of biological prior knowledge into the predictive model. Drug-induced phosphorylation does not occur independently at individual sites, but is organized through kinase–substrate relationships, protein–protein interactions, signaling pathways, and coordinated phosphosite regulation. Similarly, graph-based drug-response models that incorporate protein interaction and pathway networks have demonstrated improved predictive performance and greater mechanistic interpretability [62,63]. These observations motivate future models in which proteins, phosphosites, kinases, drugs, and pathways are represented as nodes in a heterogeneous graph, with edges encoding known protein–protein, kinase–substrate, drug–target, and phosphosite co-regulation relationships. Graph neural networks or related knowledge-graph architectures could then propagate regulatory context across these networks, allowing predictions to reflect cellular signaling organization in addition to sequence and chemical similarity. Finally, extending DrugPTM-Bench to additional PTM classes, including acetylation, ubiquitination, and methylation, as sufficiently large perturbation-resolved datasets become available would broaden the benchmark beyond its current phosphorylation-dominant setting and enable evaluation of whether modeling strategies generalize across distinct regulatory mechanisms.

## 5. Conclusions

DrugPTM-Bench establishes the first integrated benchmark dataset that simultaneously provides a standardized resource for evaluating drug-induced PTM regulation and serves as a concrete testbed for imbalanced-learning strategies in machine learning for biological settings. This resource addresses critical gaps in proteomics and pharmacology by systematically aggregating dose- and time-resolved post-translational modification (PTM) data across seven cell lines. To our knowledge, DrugPTM-Bench is the first dataset to provide dose- and time-resolved PTM regulation across multiple cell lines. By capturing dynamic PTM regulation at the systems level, our dataset enables both biological discovery and computational modeling, facilitating a deeper understanding of drug-induced PTM dynamics and enhancing predictive modeling for therapeutic applications. Our evaluation demonstrates severe performance degradation in traditional methods (random forests, decision trees) and modern neural networks with focal loss when handling extreme class imbalance inherent to PTM regulation events. DrugPTM-Bench therefore provides a foundation for evaluating more suitable imbalanced-learning strategies, including class-balanced losses based on the effective number of samples, logit-adjusted or balanced-softmax objectives that account for class-frequency bias, and uncertainty-aware approaches that reduce the influence of ambiguous regulation labels near the decision boundary. More importantly, future models may benefit from incorporating biological structure that is absent from the present baselines, such as kinase–substrate relationships, phosphosite co-regulation networks, pathway annotations, and drug–target information. Such approaches may help distinguish direct, mechanistically coherent PTM regulation from indirect or heterogeneous downstream responses. As a dual-purpose resource, DrugPTM-Bench promotes modeling of potential tasks associated with drug discovery stages and development through insights into chemically perturbed cellular phenotypes and establishes a standardized framework for benchmarking next-generation imbalanced learning algorithms in biological systems.

## Supplementary Material

Suppplementary

**Supplementary Materials:** The following supporting information can be downloaded at https://www.mdpi.com/article/10.3390/info17111079/s1, Figure S1: Threshold sensitivity analysis across all 10.7 million PTM records; Figure S2: Top-k enrichment of regulated PTM predictions across the three representative imbalance settings; Figure S3: Configurations with fixed cell line and treatment duration; Figure S4: Mean test-set MCC versus matched dose, by cell line, at four timepoints; Figure S5: RPMI8226 5x5 factorial block; Table S1: Predictive tasks enabled by DrugPTM-Bench; Table S2: Extreme class imbalance in phosphorylation site counts for prediction of PTM regulation at various dosages and time points in the benchmark dataset; Table S3: Model architectures and training hyperparameters; Table S4: Composition of the held-out test set used for benchmark evaluation; Table S5: Protein-clustered bootstrap 95% confidence intervals for MCC and AUPRC across the three representative settings; Table S6: Protein-clustered bootstrap 95% confidence intervals for the ranking-based metrics.

## Figures and Tables

**Figure 1.**
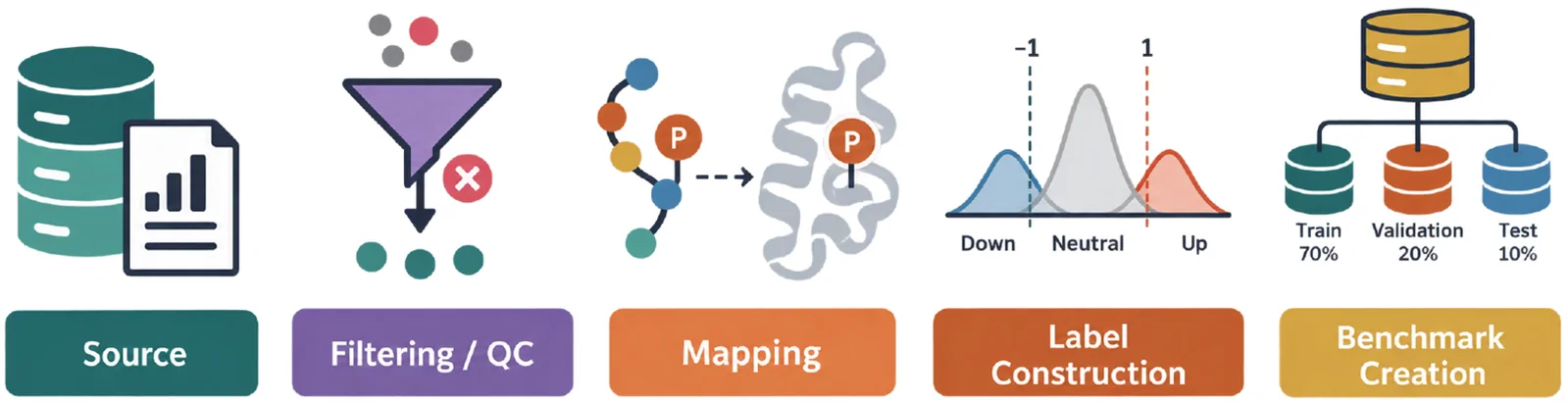
Overview of the DrugPTM-Bench curation and benchmark-construction workflow. DrugPTM-Bench is derived from processed decryptM proteomics measurements and organized through five main stages. Source: processed drug-induced PTM measurements and associated experimental metadata are obtained from decryptM. Filtering/QC: records with invalid or unusable signal-ratio values are removed prior to benchmark construction. Mapping: PTM-containing peptides are assigned to proteins using MaxQuant-derived leading-protein annotations, and peptide-local modification positions are mapped to protein-level PTM coordinates. Label construction: quantitative signal ratios are converted into three regulation classes using the operational rule log_2_(SR) < −1 for Down, −1 ≤ log_2_ (SR) ≤ 1 for Neutral, and log_2_(SR) > 1 for Up. Benchmark creation: proteins are partitioned into protein-disjoint training, validation, and test sets in a 70:20:10 ratio, with no protein overlap across splits.

**Figure 2.**
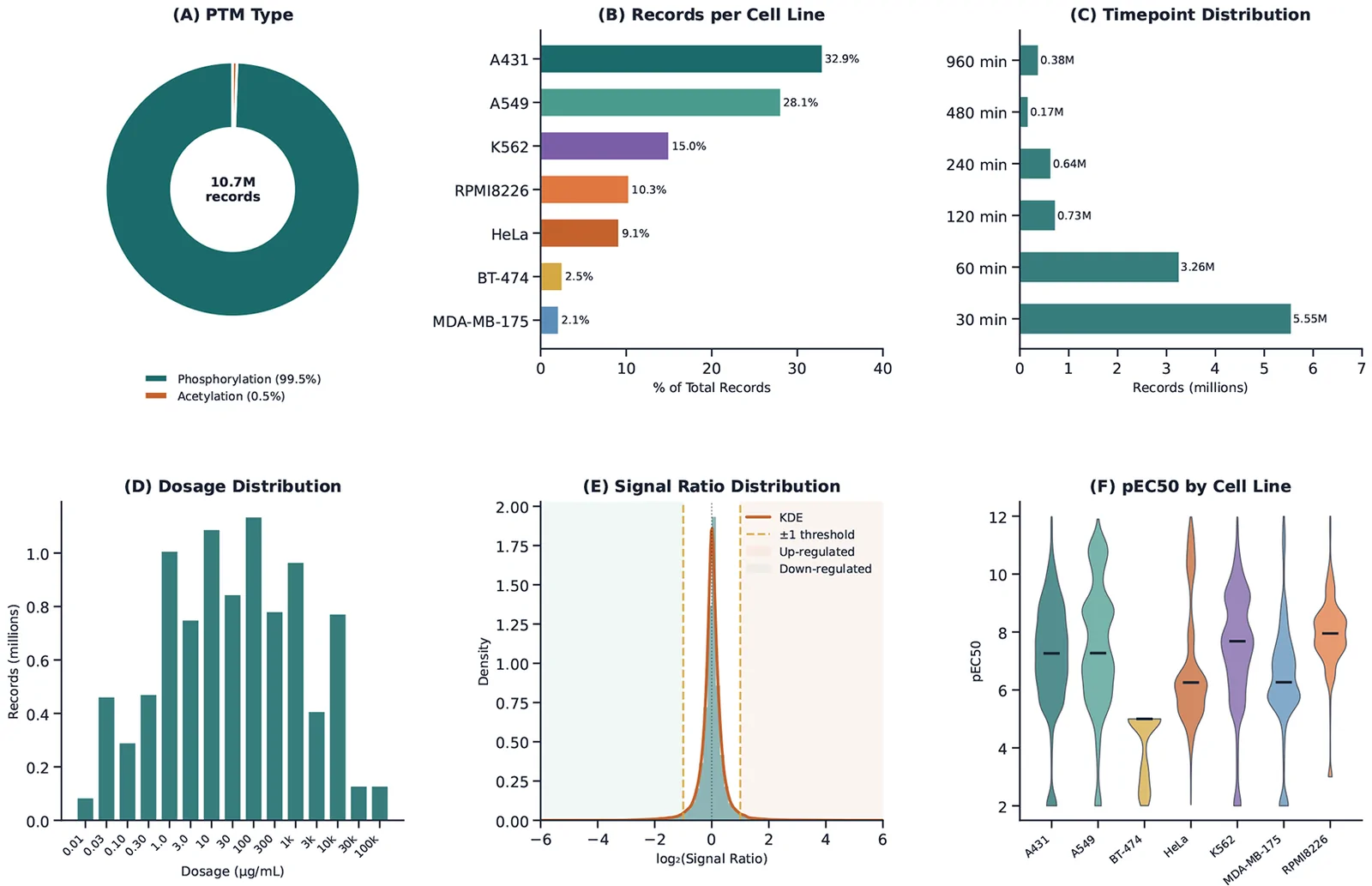
DrugPTM-Bench dataset overview. Six-panel summary of the dataset. (**A**) PTM type distribution: 99.5% phosphorylation, 0.5% acetylation, reflecting the phosphoproteomics focus of the source data. (**B**) Number of PTM measurements per cell line. (**C**) Timepoint distribution (30–960 min), with measurements concentrated at early time points. (**D**) Dosage level distribution across 15 non-zero drug concentrations (0.01–100,000 μg/mL), and untreated control measurements are retained separately. (**E**) Log_2_ signal ratio distribution across all cell lines, showing the characteristic spike at zero (neutral sites), a kernel density estimate (KDE) is overlaid to depict the bell-shaped curve. The sharp central peak indicates that the majority of PTM sites remain unchanged under treatment, presaging the severe class imbalance in the benchmark task. (**F**) pEC_50_ distribution by cell line.

**Figure 3.**
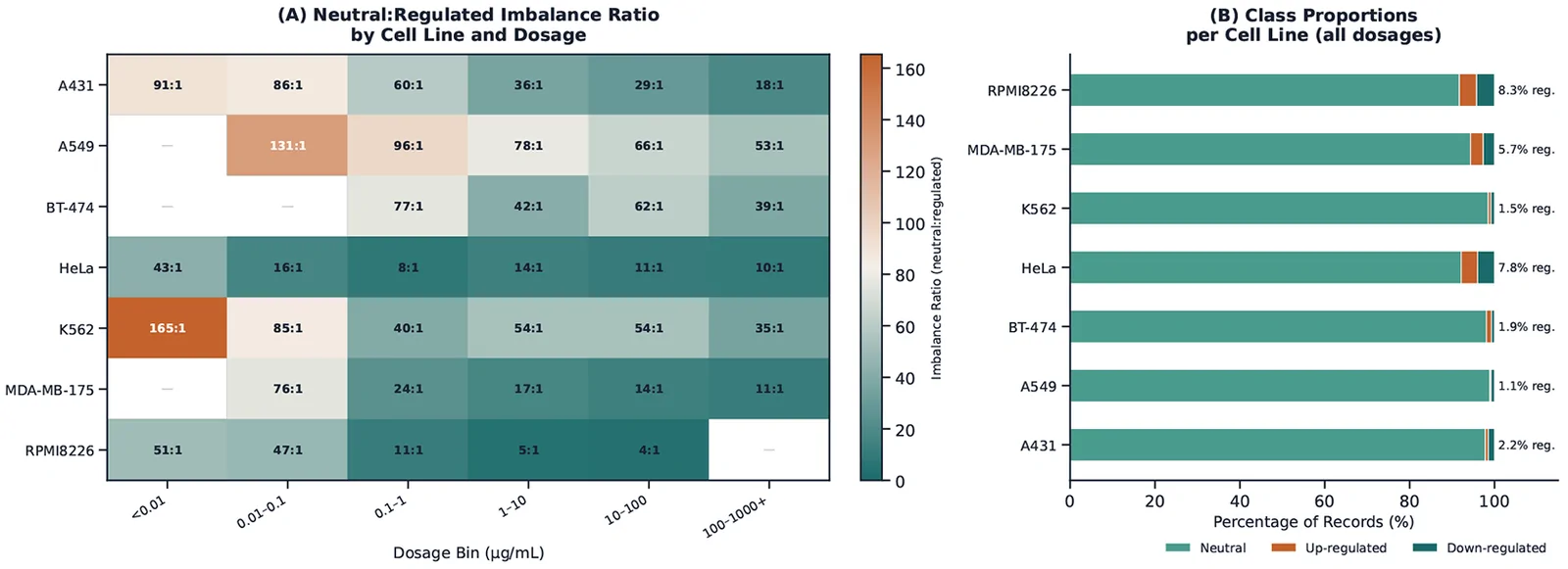
Class imbalance landscape across cell lines. (**A**) Heatmap of neutral-to-minority class ratios across cell lines and dosage levels, showing that imbalance is most extreme at low doses and varies substantially across cellular contexts. (**B**) Stacked bar chart showing the proportion of Up, Down, and Neutral PTM sites per cell line. Imbalance ratios range from 4:1 (RPMI8226) to 165:1 (K562), confirming that DrugPTM-Bench spans a wide spectrum of imbalance regimes rather than a single fixed ratio.

**Figure 4.**
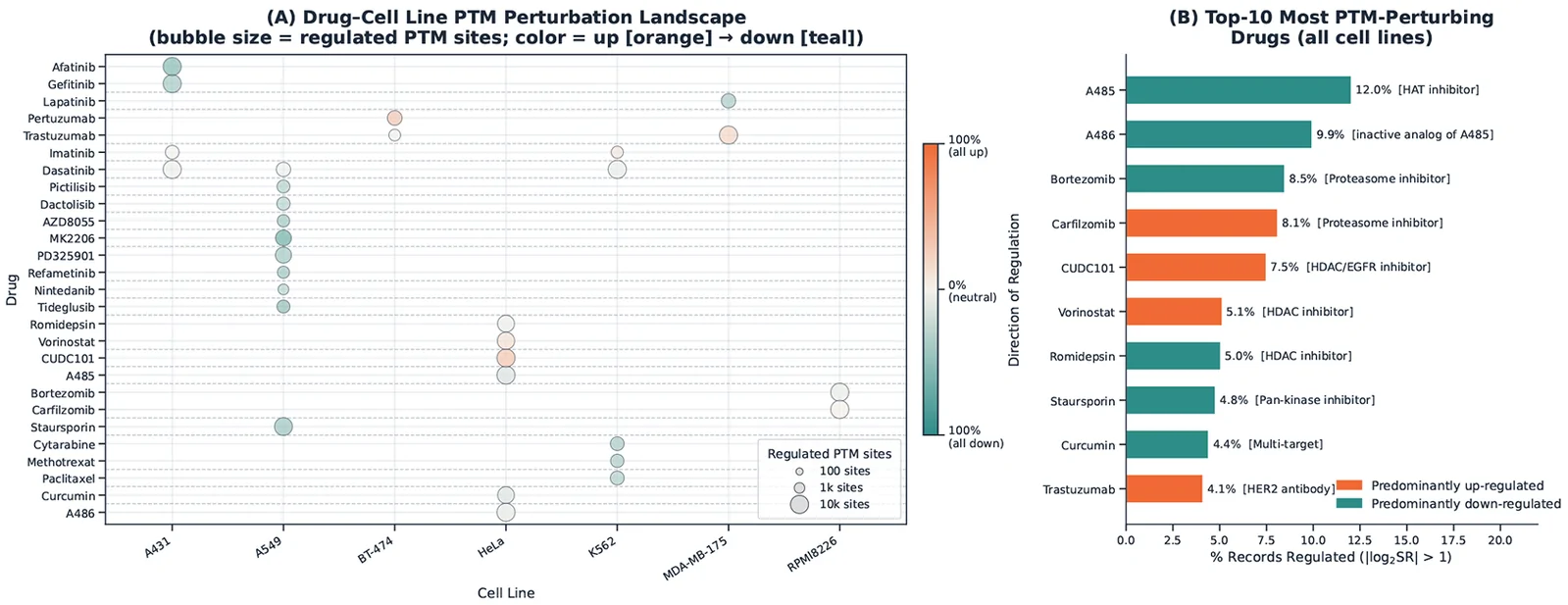
Drug-induced PTM perturbation landscape across drugs and cell lines. (**A**) Bubble plot summarizing regulated PTM responses for each drug–cell line combination after applying the benchmark regulation threshold (∣ log_2_ SR∣ > 1). Bubble size indicates the total number of regulated PTM sites (Up + Down), and bubble colour indicates the fraction of regulated sites that are up-regulated, ranging from predominantly down-regulated (teal) to predominantly up-regulated (orange). The key finding is that drug-induced PTM perturbation is highly heterogeneous across cellular contexts, with some drug–cell line combinations inducing broad PTM reprogramming while others affect only a small subset of sites. (**B**) Top 10 perturbing drugs ranked by the percentage of PTM records classified as regulated in the benchmark. The bracketed mechanism labels are literature-derived annotations added for interpretive context, including histone acetyltransferase (HAT) inhibition, histone deacetylase (HDAC) inhibition, proteasome inhibition, receptor/kinase signalling blockade, and antibody-based targeting. These annotations highlight that the most strongly perturbing compounds span diverse therapeutic mechanisms.

**Figure 5.**
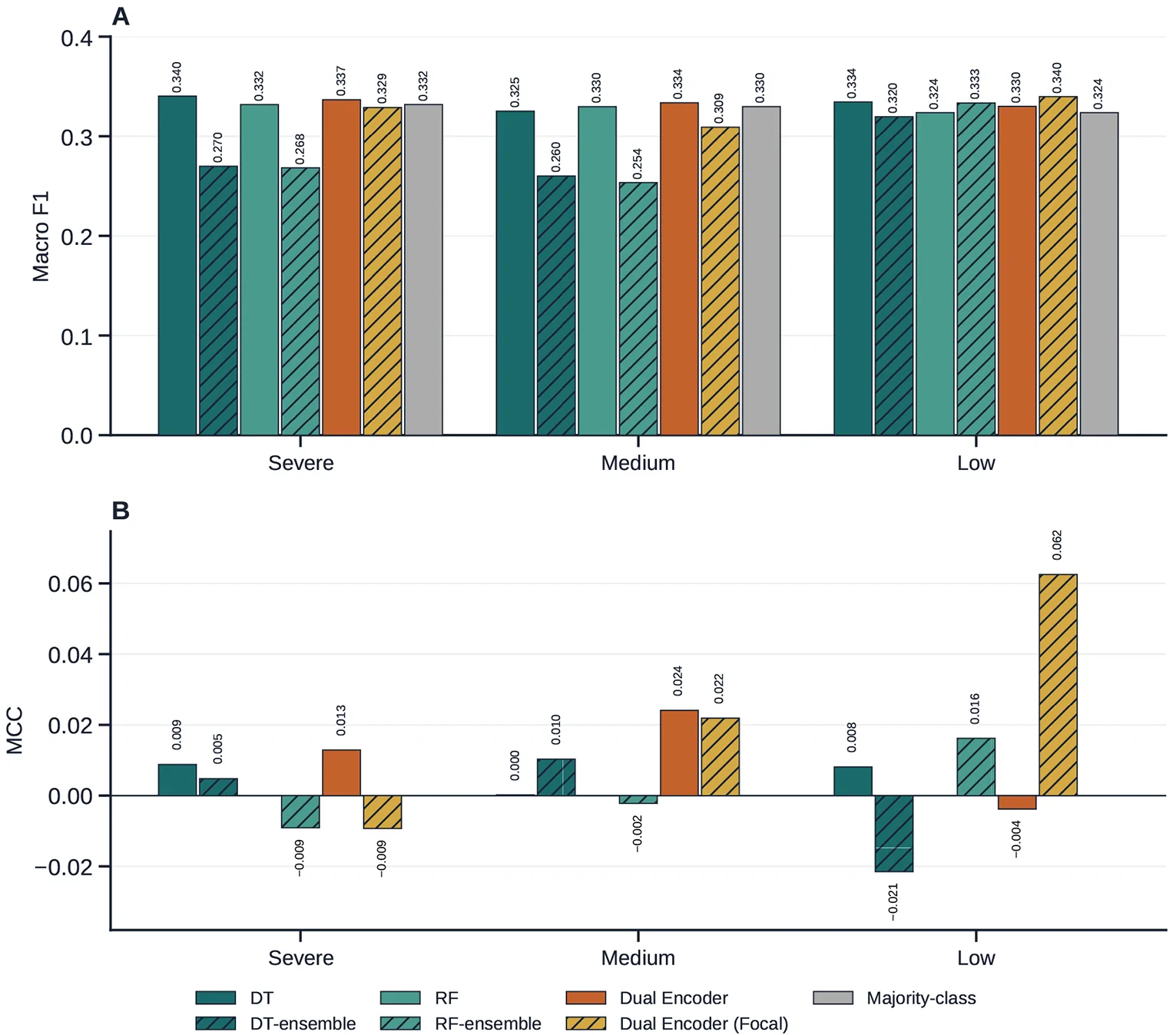
Benchmark performance across three imbalance regimes using macro-F1 and the Matthews correlation coefficient (MCC). The (**A**) reports macro-F1 and the (**B**) reports MCC for the ML and DL baselines. Macro-F1 remains relatively similar across several settings despite major differences in minority-class recoverability, whereas MCC more clearly distinguishes meaningful performance under imbalance. The black bar shows the trivial model of majority-class prediction, while the Macro F1 remains competitive of the various baselines with the trivial classifier, the MCC clearly shows the inability to predict the minority classes.

**Figure 6.**
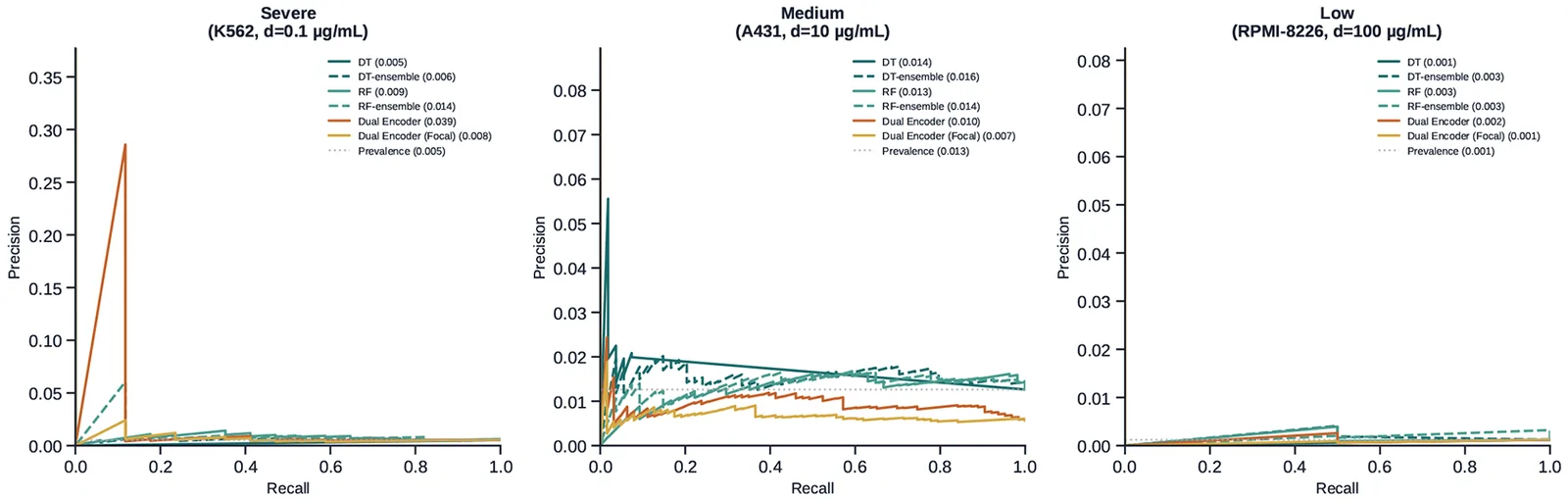
Precision-recall curves for the Up and Down classes across the three representative settings (Severe, Medium, Low). Each curve corresponds to one of the six baseline models. The horizontal dashed line indicates the class prevalence (random-ranking baseline). In the Severe setting, the Dual Encoder’s PR curve for the Up class rises well above the prevalence line at low recall, consistent with its high prevalence-relative AUPRC (7.40) despite near-zero MCC

**Figure 7.**
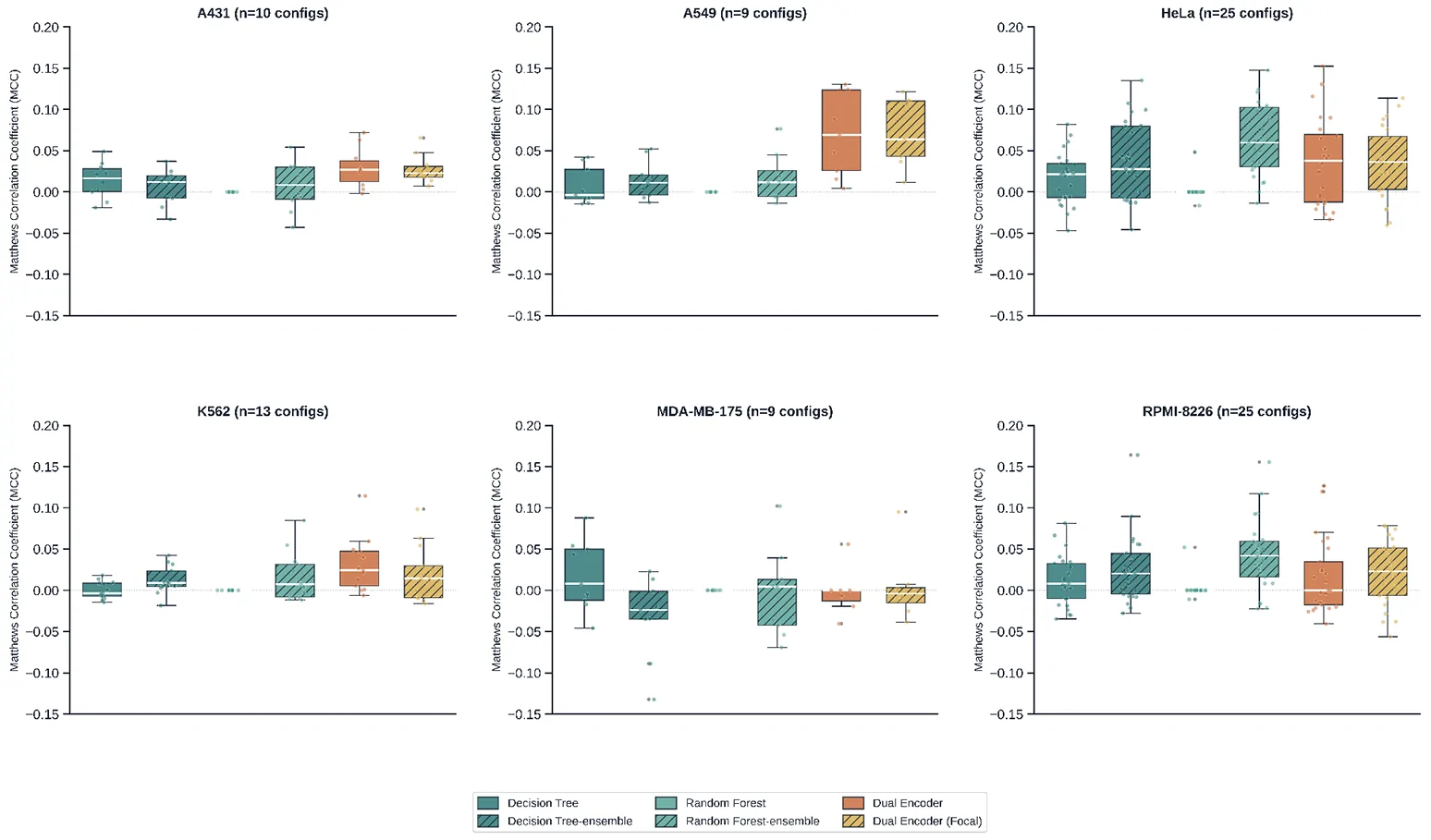
MCC boxplot distributions by model and cell line across the 91 unique configurations of DrugPTM-Bench. Each panel shows one cell line, with the number of configurations given in parentheses. The key finding is that MCC values are low across all models and configurations (range: −0.13 to 0.16), and the win distribution tracks the underlying imbalance regime rather than favoring a single model architecture

**Table 1.** Comparison of different PTM datasets with DrugPTM-Bench. Existing resources are primarily static site catalogs, whereas DrugPTM-Bench additionally captures drug, dose, time, regulation labels, and cell-type context (Δ denotes partial PTM regulation info).

Dataset	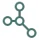 PTM Sites	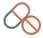 Drug Dependency	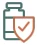 Dosage Dependency	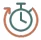 Time Dependency	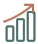 PTM Regulation	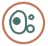 Cell Type Context
**DrugPTM-Bench (Ours)**	**✓**	**✓**	**✓**	**✓**	**✓**	**✓**
dbPTM [23]	**✓**	**✗**	**✗**	**✗**	Δ	**✗**
BioGRID [24]	**✓**	**✗**	**✗**	**✗**	**✗**	**✗**
PhosphoSitePlus [25]	**✓**	**✗**	**✗**	**✗**	**✗**	**✗**
EPSD 2.0 [34]	**✓**	**✗**	**✗**	**✗**	**✗**	**✗**
ActiveDriverDB [35]	**✓**	**✗**	**✗**	**✗**	**✗**	**✗**
Phospho.ELM [33]	**✓**	**✗**	**✗**	**✗**	**✗**	**✗**
UniProtKB [36]	**✓**	**✗**	**✗**	**✗**	**✗**	**✗**

**Table 2.** Benchmark performance across imbalance regimes. Ensemble models denote training on rebalanced subsets. The Majority-class row shows the trivial baseline that always predicts Neutral. Metrics are grouped by class to highlight class-specific performance. Arrows indicate that higher values are better (↑). Best value per class and metric within each imbalance regime is shown in bold.

Imbalance	Model Family	Model	Neutral Class (↑)			Up Class (↑)			Down Class (↑)		
			Recall	Precision	AUPRC	Recall	Precision	AUPRC	Recall	Precision	AUPRC
Severe	ML	DT	0.974	0.991	0.991	0.000	0.000	0.005	0.083	**0.025**	0.008
		RF	**1.000**	0.991	**0.993**	0.000	0.000	0.009	0.000	0.000	**0.018**
		DT-ensemble	0.653	**0.991**	0.990	0.235	**0.005**	0.006	**0.167**	0.007	0.006
		RF-ensemble	0.633	0.990	0.991	**0.294**	0.005	0.014	0.083	0.014	0.010
	DL	Dual Encoder	0.979	0.991	0.989	0.000	0.000	**0.039**	0.083	0.015	0.004
		Dual Encoder (Focal)	0.983	0.991	0.988	0.000	0.000	0.008	0.000	0.000	0.004
	–	Majority-class	1.000	0.991	0.991	0.000	0.000	0.005	0.000	0.000	0.004
Medium	ML	DT	0.897	0.979	0.979	0.074	**0.021**	0.014	0.027	0.004	0.009
		RF	**1.000**	0.979	0.984	0.000	0.000	0.013	0.000	0.000	0.010
		DT-ensemble	0.588	0.980	0.985	0.296	0.015	**0.016**	**0.162**	0.009	0.011
		RF-ensemble	0.592	0.979	0.984	**0.333**	0.012	0.014	0.000	0.000	0.009
	DL	Dual Encoder	0.925	0.985	**0.989**	0.016	0.014	0.010	0.126	**0.018**	**0.013**
		Dual Encoder (Focal)	0.801	**0.986**	0.988	0.206	0.011	0.007	0.117	0.013	0.011
	–	Majority-class	1.000	0.979	0.979	0.000	0.000	0.013	0.000	0.000	0.009
Low	ML	DT	0.944	0.944	0.944	**0.000**	**0.000**	0.001	0.056	0.062	0.057
		RF	**1.000**	0.944	0.954	**0.000**	**0.000**	0.003	0.000	0.000	0.061
		DT-ensemble	0.878	0.942	0.934	**0.000**	**0.000**	**0.003**	0.067	0.040	0.049
		RF-ensemble	0.986	0.944	**0.955**	**0.000**	**0.000**	0.003	0.022	0.087	0.060
	DL	Dual Encoder	0.887	0.942	0.945	**0.000**	**0.000**	0.002	0.089	0.067	0.085
		Dual Encoder (Focal)	0.790	**0.951**	0.944	**0.000**	**0.000**	0.001	**0.233**	**0.118**	**0.089**
	–	Majority-class	1.000	0.944	0.944	0.000	0.000	0.001	0.000	0.000	0.055

**Table 3.** MCC performance comparison across models. Win counts are based on 91 unique configurations (cell line × timepoint × dosage). Ties are split equally.

Model	Number of Configs with Best MCC	Mean MCC
Decision Tree	8.0	0.0119
Decision Tree-ensemble	11.5	0.0175
Random Forest	3.5	0.0009
Random Forest-ensemble	29.5	0.0345
Dual Encoder	22.0	0.0296
Dual Encoder (Focal)	16.5	0.0292

## Data Availability

The data presented in this study are openly available at https://figshare.com/s/76f0986604b74c29fe89 (accessed on 26 October 2026). The protein OOD splits used in experiments are present at https://figshare.com/s/f56a376fb36fb829d2a3 (accessed on 26 October 2026).
