# Supplementary material for: DrugPTM-Bench: A Large-Scale Dataset for Predictive Modeling of Drug-Induced Cell Type-Specific Protein Post-Translational Modifications
Source: Information (Basel). Author manuscript; Available in PMC 2026 Nov 7. (PMC13742136; doi:10.3390/info17111079)
Supplement: Supplementary file 1 [file NIHMS2220693-supplement-Suppplementary.pdf]

Supplementary Material

DrugPTM-Bench: A Large-Scale Dataset for Predictive Modeling of Drug-Induced Cell Type-Specific Protein Post-Translational Modifications

Amitesh Badkul[1,*], Li Xie[2], Mohammadsadeq Mottaqi[3], and Lei Xie[1,4]

[1]Ph.D. Program in Computer Science, The Graduate Center, The City University of New York, New York, NY, USA

[2]Department of Computer Science, Hunter College, The City University of New York, New York, NY, USA

[3]Ph.D. Program in Biochemistry, The Graduate Center, The City University of New York, New York, NY, USA

[4]School of Pharmacy and Pharmaceutical Sciences and Center for Drug Discovery, Northeastern University, Boston, MA, USA

[*]Corresponding author: abadkul@gc.cuny.edu

Additional Predictive Tasks Enabled by DrugPTM-Bench

While the primary benchmark task in this work is PTM regulation classification (T1), the multi-dimensional structure of DrugPTM-Bench. The benchmark encompasses pEC50 potency values, full dose-response curves, drug perturbation fingerprints across thousands of sites, and site-level sequence context enabling a family of additional drug discovery relevant tasks. These tasks are formally defined here to establish a community roadmap and invite future contributions. They are not benchmarked in the present work. Each task fills a gap not addressed by existing benchmarks.

T2: pEC50 Potency Regression

In decryptM [13], each PTM site $\times$ drug combination with a measurable dose-response is fitted across 11 drug concentrations to yield a pEC50 value. The negative $\log_{10}$ of the molar concentration at which the signal ratio reaches half its maximum effect. The T2 task is to predict this pEC50 from protein sequence context, drug structure, cell line identity, bypassing the need to run the full 11-point dose-response assay. This is fundamentally different from the canonical drug-target affinity benchmarks Davis and KIBA [12], which predict thermodynamic binding affinity (K_d or K_i) measured in cell-free biochemical assays. The pEC50 in DrugPTM-Bench is a *functional cellular potency*: it reflects not only direct binding but also downstream pathway buffering, feedback regulation, and cell-line-specific signaling wiring [13]. A drug can have high binding affinity for a kinase yet low pEC50 on a downstream phosphosite if the pathway is buffered by compensatory mechanisms, a distinction that cell-free affinity measurements cannot capture. From a drug discovery perspective, a model that accurately predicts pEC50 at PTM-site resolution could prioritize which drug-site pairs to validate experimentally, reducing the cost of dose-response phosphoproteomics by an order of magnitude. An important limitation is that pEC50 values are only defined for regulated sites ($\sim$47,000 site $\times$ drug pairs in DrugPTM-Bench), making the training set smaller than Davis or KIBA, smaller data regimes are very common in computational drug discovery.

T3: Mechanism-of-Action Prediction from PTM Profile

Drugs with the same mechanism of action perturb overlapping sets of PTM sites: kinase inhibitors suppress phosphorylation of their direct substrates and downstream effectors, HDAC inhibitors broadly alter histone and non-histone acetylation, and proteasome inhibitors trigger widespread phosphorylation changes through proteostatic stress [13, 3]. A PTM perturbation fingerprint, the vector of signal ratios across all detected sites for a given drug in a given cell line, is therefore mechanistically closer to drug action than transcriptomic signatures, because PTMs are the direct molecular readout of kinase, phosphatase, acetyltransferase, and deacetylase activity rather than a downstream transcriptional consequence. The T3 task is to predict a drug's mechanism class from its PTM fingerprint. Existing MoA prediction benchmarks, including LINCS L1000 [11], MoAble [5], MOASL [6], and the PANACEA DREAM Challenge [2], all use transcriptomic perturbation signatures as input, and none use PTM-level profiles. Mitchell et al. [9] profiled 875 compounds by quantitative proteomics (protein expression, not PTM) and demonstrated MoA clustering, but PTM resolution was not exploited. With 27 drugs spanning eight mechanism classes in DrugPTM-Bench, T3 is a tractable but non-trivial classification problem that could reveal whether PTM fingerprints encode MoA information not captured by transcriptomics.

T4: Drug-Specific PTM Site Sensitivity Ranking

Mass spectrometry-based phosphoproteomics experiments routinely detect thousands of PTM sites per condition, but experimental follow-up (mutagenesis, functional assays, structural studies) can only be performed on a handful of sites [10]. A computational model that ranks PTM sites by their predicted sensitivity to a specific drug would directly reduce this experimental bottleneck by prioritizing the most drug-responsive sites for validation. Existing PTM prioritization methods rank sites by intrinsic functional importance: Beltrão et al. [1] used evolutionary conservation and structural context. Ochoa et al. [10] integrated 6,801 proteomics experiments to build a functional landscape of the human phosphoproteome. Lastly, Kennedy et al. [8] developed PTM-centric base editor screens to assess phosphosite functionality at scale experimentally. All of these approaches are drug-agnostic, they rank sites by their general biological importance, not by their sensitivity to a particular perturbation. DrugPTM-Bench enables the first drug-specific PTM site sensitivity ranking benchmark, where the input is a drug fingerprint paired with a protein sequence and all its candidate PTM site contexts, and the output is a ranked list of sites by predicted |signal ratio|. The Johnson et al. kinome substrate specificity atlas [7], which maps the sequence preferences of over 300 human Ser/Thr kinases, provides a natural source of sequence-level priors for T4 models targeting phosphorylation sites. Appropriate evaluation metrics for T4 include normalized discounted cumulative gain (NDCG) and Spearman correlation between predicted and observed |signal ratio| rankings per drug-protein pair.

Table S1. Predictive tasks enabled by DrugPTM-Bench. T1 is benchmarked in this work. T2-T4 are defined here as future benchmark tasks. All tasks are formulated at PTM-site resolution.

Task	Setting	Inputs	Target	Why this is new
**T1. PTM Regulation Classification** *(benchmarked in this work)*	3-class classification	PTM-site sequence context, drug identity, dosage, and cell line	Neutral / Up / Down	No existing benchmark jointly models drug identity, dosage, cell context, and direction of PTM regulation at site resolution ([13, 4])
**T2. pEC50 Potency Regression**	Regression	PTM-site sequence context, drug identity, and cell line	Site-level pEC50	Benchmarks such as Davis and KIBA focus on biochemical binding affinity, whereas this task asks for functional cellular potency at PTM-site resolution, which is a different prediction problem ([12, 13])
**T3. Mechanism-of-Action Prediction from PTM Profiles**	Multi-class classification	Drug-induced PTM response profile within a given cell line	Drug mechanism class	Existing benchmarks such as LINCS L1000 and PANACEA are based on transcriptomic response signatures. This task instead uses PTM-level perturbation profiles ([9, 2, 13])
**T4. Drug-Specific PTM Site Sensitivity Ranking**	Learning-to-rank	Drug identity, protein context, and candidate PTM sites on the same protein	Ranked PTM sites by predicted response magnitude	Existing PTM prioritization methods rank sites by intrinsic importance, such as conservation or network relevance, rather than by drug-specific sensitivity ([10, 1, 8, 7])

PTM Event Regulation Distribution (T1 data distribution)

Table S2: Extreme class imbalance in phosphorylation site counts for prediction of PTM regulation at various dosages and time points in the benchmark dataset.

Cell line	Dosage (μg)	Timepoint (min)	All	Neutral	Up	Down	Regulated (%)	Neutral: Regulated	N proteins	N drugs
A431	0	30	174758	174758	–	–	0.00	–	–	
A431	0.03	30	174661	172673	907	1081	1.14	86.9	3658	
A431	0.3	30	174646	172437	837	1372	1.26	78.1	3656	
A431	1	30	174697	172871	1075	751	1.05	94.7	3658	
A431	3	30	174692	171893	1385	1414	1.60	61.4	3659	
A431	10	30	174683	171866	1256	1561	1.61	61.0	3658	
A431	30	30	174709	170714	1456	2539	2.29	42.7	3657	
A431	100	30	174684	169190	1602	3892	3.15	30.8	3659	
A431	300	30	174686	168631	1727	4328	3.47	27.8	3659	
A431	1000	30	174675	168820	1586	4269	3.35	28.8	3658	
A431	10000	30	174686	165355	2246	7085	5.34	17.7	3658	
**A431 (mean)**	—	—	**174682**	**170445**	**1408**	**2829**	**2.43**	**40.2**	**3658**	**4**
A549	0	60	274619	273817	397	405	0.29	341.4	–	
A549	1	60	157947	156730	400	817	0.77	128.8	2948	
A549	3	60	126852	125798	351	703	0.83	119.4	2928	
A549	10	60	157819	156034	584	1201	1.13	87.4	2947	
A549	30	60	126760	125102	519	1139	1.31	75.5	2927	
A549	100	60	157900	155684	747	1469	1.40	70.3	2948	
A549	300	60	126757	124875	577	1305	1.48	66.4	2926	
A549	1000	60	126762	124683	586	1493	1.64	60.0	2928	
A549	3000	60	126677	124502	692	1483	1.72	57.2	2927	
A549	10000	60	126635	124152	799	1684	1.96	50.0	2927	
**A549 (mean)**	—	—	**137123**	**135284**	**584**	**1255**	**1.34**	**73.6**	**2934**	**10**
HeLa	0	30	20367	20367	–	–	0.00	–	–	
HeLa	0	240	45539	42274	1541	1724	7.17	12.9	–	
HeLa	0	960	12335	12315	12	8	0.16	615.8	–	
HeLa	0.03	960	6144	6097	23	24	0.76	129.7	1476	
HeLa	0.1	960	6146	6090	21	35	0.91	108.8	1477	
HeLa	0.3	960	6144	6071	33	40	1.19	83.2	1478	
HeLa	1	960	6154	5822	96	236	5.39	17.5	1482	
HeLa	3	960	6148	5907	101	140	3.92	24.5	1479	
HeLa	10	30	20351	17472	1017	1862	14.15	6.1	2057	
HeLa	10	960	6150	5740	167	243	6.67	14.0	2057	
HeLa	30	30	20357	17999	1126	1232	11.58	7.6	2057	
HeLa	30	240	6247	6078	30	139	2.71	36.0	2057	
HeLa	30	960	6142	5724	176	242	6.81	13.7	2057	
HeLa	100	30	20360	19062	806	492	6.38	14.7	2056	
HeLa	100	240	22764	22193	293	278	2.51	38.9	2056	
HeLa	100	960	6141	5644	180	317	8.09	11.4	2056	
HeLa	300	30	20351	18250	944	1157	10.32	8.7	2057	
HeLa	300	240	22726	22015	341	370	3.13	31.0	2057	
HeLa	1000	30	20355	16637	1403	2315	18.27	4.5	2057	
HeLa	1000	240	22760	22188	339	233	2.51	38.8	2057	
HeLa	3000	30	20313	19765	194	354	2.70	36.1	2055	
HeLa	3000	240	22801	22249	314	238	2.42	40.3	2055	
HeLa	10000	30	20355	14766	2218	3371	27.46	2.6	2057	
HeLa	10000	240	22806	21873	461	472	4.09	23.4	2057	
HeLa	30000	30	20347	16846	1278	2223	17.21	4.8	2056	
HeLa	30000	240	22805	21887	510	408	4.03	23.8	2056	
HeLa	100000	30	20339	18876	634	829	7.19	12.9	2056	
HeLa	100000	240	22783	21621	691	471	5.10	18.6	2056	
**HeLa (mean)**	—	—	**15920**	**14675**	**536**	**709**	**7.82**	**11.8**	**1941**	**6**
K562	0	30	83950	83950	–	–	0.00	–	–	
K562	0.01	30	44385	44237	103	45	0.33	298.9	2367	
K562	0.03	30	68663	68358	188	117	0.44	224.1	2801	
K562	0.1	30	49108	48702	213	193	0.83	120.0	2689	
K562	0.3	30	73383	72887	322	174	0.68	146.9	3072	

Continued on next page

Table S2 – continued from previous page

Cell line	Dosage (µg)	Timepoint (min)	All	Neutral	Up	Down	Regulated (%)	Neutral: Regulated	N proteins	N drugs
K562	1	30	83653	82543	668	442	1.33	74.4	3175	
K562	3	30	83665	80915	1770	980	3.29	29.4	3174	
K562	10	30	83602	82381	567	654	1.46	67.5	3177	
K562	30	30	83660	82311	552	797	1.61	61.0	3184	
K562	100	30	83625	82191	505	929	1.71	57.3	3177	
K562	300	30	39301	38737	323	241	1.44	68.7	2767	
K562	1000	30	39329	38812	230	287	1.31	75.1	2768	
K562	3000	30	10248	10086	97	65	1.58	62.3	1547	
K562	10000	30	34540	33761	442	337	2.26	43.3	2538	
**K562 (mean)**	—	—	**59782**	**58917**	**460**	**405**	**1.45**	**68.1**	**2803**	**5**
MDA-MB-175	0	120	5395	5395	–	–	0.00	–	–	
MDA-MB-175	1	120	5392	5336	31	25	1.04	95.3	1215	
MDA-MB-175	3	120	5393	5327	24	42	1.22	80.7	1215	
MDA-MB-175	10	120	5392	5323	13	56	1.28	77.1	1215	
MDA-MB-175	30	120	5392	5326	22	44	1.22	80.7	1215	
MDA-MB-175	100	120	5394	5349	7	38	0.83	118.9	1215	
MDA-MB-175	300	120	5390	5082	51	257	5.71	16.5	1214	
MDA-MB-175	1000	120	5391	5117	36	238	5.08	18.7	1215	
MDA-MB-175	3000	120	5390	4927	91	372	8.59	10.6	1214	
MDA-MB-175	10000	120	5388	4724	193	471	12.32	7.1	1214	
**MDA-MB-175 (mean)**	—	—	**5391**	**5168**	**52**	**171**	**4.14**	**23.1**	**1215**	**2**
RPMI8226	0	60	24768	24768	–	–	0.00	–	–	
RPMI8226	0	120	23524	23524	–	–	0.00	–	–	
RPMI8226	0	240	20814	20814	–	–	0.00	–	–	
RPMI8226	0	480	17776	17776	–	–	0.00	–	–	
RPMI8226	0	960	26439	26439	–	–	0.00	–	–	
RPMI8226	0.1	60	24758	23782	286	690	3.94	24.4	2114	
RPMI8226	0.1	120	23504	23091	145	268	1.76	55.9	2114	
RPMI8226	0.1	240	20784	20629	88	67	0.75	133.1	2114	
RPMI8226	0.1	480	17764	17577	136	51	1.05	94.0	2114	
RPMI8226	0.1	960	26384	25873	285	226	1.94	50.6	2114	
RPMI8226	1	60	24754	23732	72	950	4.13	23.2	2113	
RPMI8226	1	120	23502	22969	137	396	2.27	43.1	2113	
RPMI8226	1	240	20791	20653	90	48	0.66	149.7	2113	
RPMI8226	1	480	17764	17483	247	34	1.58	62.2	2113	
RPMI8226	1	960	26402	25902	344	156	1.89	51.8	2113	
RPMI8226	10	60	24755	24164	42	549	2.39	40.9	2112	
RPMI8226	10	120	23515	22862	242	411	2.78	35.0	2112	
RPMI8226	10	240	20786	20407	276	103	1.82	53.8	2112	
RPMI8226	10	480	17762	16989	681	92	4.35	22.0	2112	
RPMI8226	10	960	26431	18988	4320	3123	28.16	2.6	2112	
RPMI8226	100	60	24758	23638	63	1057	4.52	21.1	2113	
RPMI8226	100	120	23513	22870	392	251	2.73	35.6	2113	
RPMI8226	100	240	20800	19226	1227	347	7.57	12.2	2113	
RPMI8226	100	480	17764	14219	2101	1444	19.96	4.0	2113	
RPMI8226	100	960	26413	12412	6414	7587	53.01	0.9	2113	
RPMI8226	1000	60	24761	23651	294	816	4.48	21.3	2113	
RPMI8226	1000	120	23515	21892	896	727	6.90	13.5	2113	
RPMI8226	1000	240	20802	18564	1666	572	10.76	8.3	2113	
RPMI8226	1000	480	17766	13761	2321	1684	22.54	3.4	2113	
RPMI8226	1000	960	26416	11652	6594	8170	55.89	0.8	2113	
**RPMI8226 (mean)**	—	—	**22647**	**20279**	**1174**	**1193**	**10.45**	**8.6**	**2113**	**2**

Threshold Sensitivity Analysis

To assess how the choice of regulation threshold affects benchmark difficulty, we recomputed class labels at five alternative thresholds ($|\log_2(\mathrm{SR})| > T$ for $T \in \{0.5, 0.75, 1.0, 1.5, 2.0\}$) across all 10.7 million PTM records. Figure S1 shows the resulting class distributions and imbalance ratios. At the most permissive threshold ($T = 0.5$), the mean regulated fraction across configurations is 15.9% with a mean imbalance ratio of 7.9:1, producing a substantially more balanced benchmark. At the default threshold ($T = 1.0$), the regulated fraction drops to 5.0% with a mean imbalance of 52.1:1. At the most stringent threshold ($T = 2.0$), only 1.1% of sites are regulated and the mean imbalance ratio exceeds 797:1, making the classification task nearly degenerate. This analysis confirms that the threshold choice is an important determinant of benchmark difficulty: a lower threshold would make the task more tractable but may include noisy or biologically insignificant responses, while a higher threshold yields cleaner labels but extreme imbalance. The $T = 1.0$ threshold represents a pragmatic balance between biological signal and class separability and is used in literature as well.

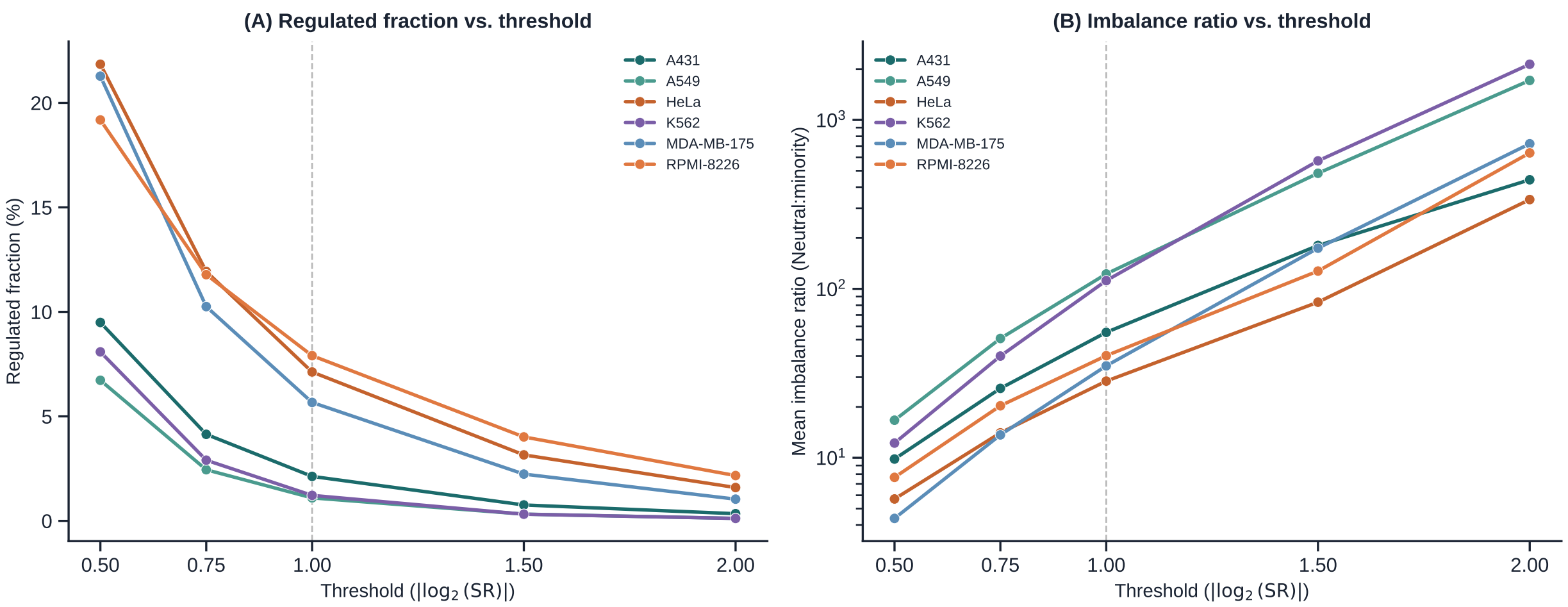

Figure S1. Threshold sensitivity analysis across all 10.7 million PTM records. (A) Regulated fraction (Up + Down) as a function of the classification threshold $|\log_2(\mathrm{SR})| > T$, shown per cell line. (B) Mean imbalance ratio (Neutral:minority) on a log scale. The dashed vertical line marks the default threshold ($T = 1.0$).

Table S3. Model architectures and training hyperparameters. ML models were implemented using scikit-learn. Unless otherwise indicated, default hyperparameters were used.

Family	Model / Component	Hyperparameter / Design choice	Value
ML	Decision Tree (DT)	Training set	Full unbalanced training set
		Criterion	Gini
	Random Forest (RF)	Training set	Full unbalanced training set
		Number of estimators	100
		Criterion	Gini
	DT-ensemble	Balanced subsets	Neutral-class partitions + all minority examples
		Aggregation	Majority vote
	RF-ensemble	Balanced subsets	Neutral-class partitions + all minority examples
		Aggregation	Majority vote
DL	Dual Encoder	Architecture	Separate protein and drug MLP branches
		Hidden dimension	256
		Dropout	0.2
	CE variant	Loss	Cross-entropy
	Focal variant	Loss	Focal loss ($\gamma = 2.0$)
	Shared training	Optimizer	Adam
		Learning rate	1×10^{-3}
		Batch size	64
		Maximum epochs / early stopping	50 / patience 10 on validation loss

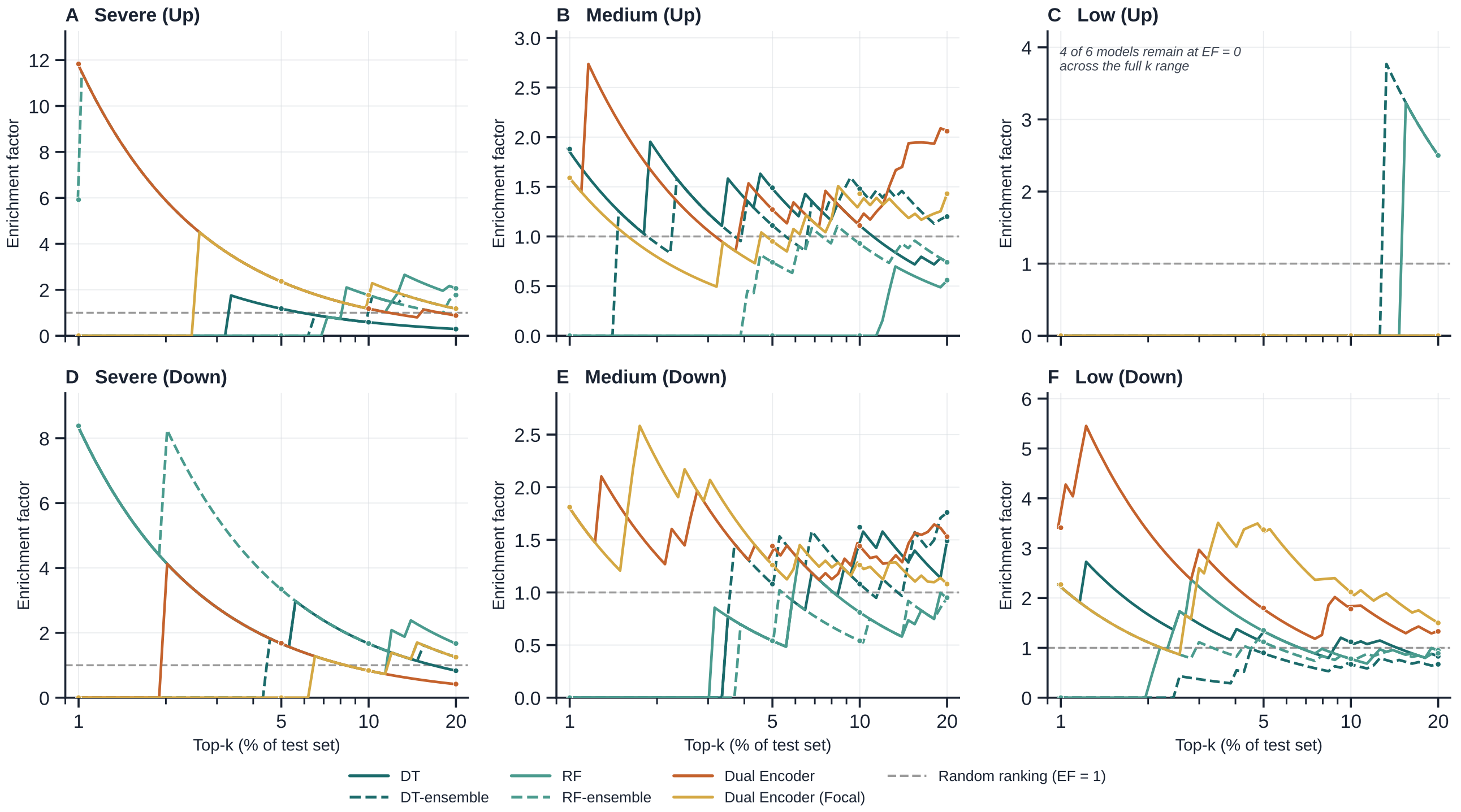

Figure S2. Top-k enrichment of regulated PTM predictions across the three representative imbalance settings. Enrichment factor (EF) is shown as a function of the top k% of test-set predictions ranked by the predicted probability of the target class, with $EF = 1$ representing random ranking. Panels (A–C) show enrichment for the Up class under the Severe, Medium, and Low settings, respectively, while (D–F) show the corresponding results for the Down class. Curves are shown for DT, DT-ensemble, RF, RF-ensemble, Dual Encoder, and Dual Encoder with focal loss. Values above $EF = 1$ indicate enrichment of true regulated sites relative to their prevalence in the test set, with the strongest enrichment generally concentrated among the highest-confidence predictions.

Controlled context analysis

In this section, we attempt to perform a controlled analysis of the context for the benchmark by asking whether a specific biological component is responsible for the imbalance and the subsequent performance of the compounds. This is done by varying atmost one component of the experiments - dosage, treatment duration (time point), and cell line.

Dose-controlled analysis

We fixed cell line and treatment time and examined dose series within the same experimental context. Increasing dosage generally shifted the observed class distribution toward a larger fraction of regulated PTM events as seen in Fig. S3, thereby reducing the neutral-to-regulated ratio. However, these dose-associated non-monotonic changes in imbalance were naturally not accompanied by a consistent monotonic change in MCC across baseline models.

Time-controlled analysis

We next examined treatment-duration effects while holding the cell line and dosage fixed. This comparison is supported most extensively by RPMI8226, which contains a complete dose-time grid. Treatment duration did not produce a uniform shift in the observed class distribution across cellular contexts. In RPMI8226, longer treatment durations generally reduced the neutral-to-regulated ratio, however, this was also applicable only to the higher dosage observations as seen in the heat map of Fig. S4. Importantly, these time-associated changes in class composition were not accompanied by a consistent change in predictive performance. Across the available matched comparisons, MCC did not vary monotonically with treatment duration, nor did configurations with lower imbalance consistently yield higher MCC. Because treatment duration changes the underlying signaling state, including direct target-proximal responses, downstream pathway propagation, feedback regulation, and adaptive responses, these comparisons do not isolate an independent effect of class imbalance. Rather, they show that the relationship between the observed class distribution and predictive performance depends on the temporal biological state represented by the measurement. Such temporal heterogeneity is expected in phosphoproteomic data because early and late measurements can represent qualitatively different signaling states rather than simply stronger or weaker versions of the same response.

Cell-line-controlled analysis

Finally, we examined cell-line effects by matching treatment time and nominal dosage across cell lines. Despite identical nominal experimental conditions, the resulting class distributions differed substantially across cellular backgrounds. For example, at matched timepoints and doses, K562 and A549 generally exhibited substantially higher neutral-to-regulated ratios than HeLa or RPMI8226. Thus, the observed imbalance is not determined solely by dose or treatment duration, but also reflects cell-specific differences in the underlying phosphoproteomic response. Fig. S5 compares mean MCC across cell lines at matched timepoints and doses. At 30 min, HeLa generally achieves higher MCC than A431 and K562 across the shared dose range, although K562 shows a sharp local increase around $\log_{10}$ dose ≈ 2.5. At 60 min, the relative ordering between A549 and RPMI8226 changes with dose: RPMI8226 performs substantially better at $\log_{10}$ dose

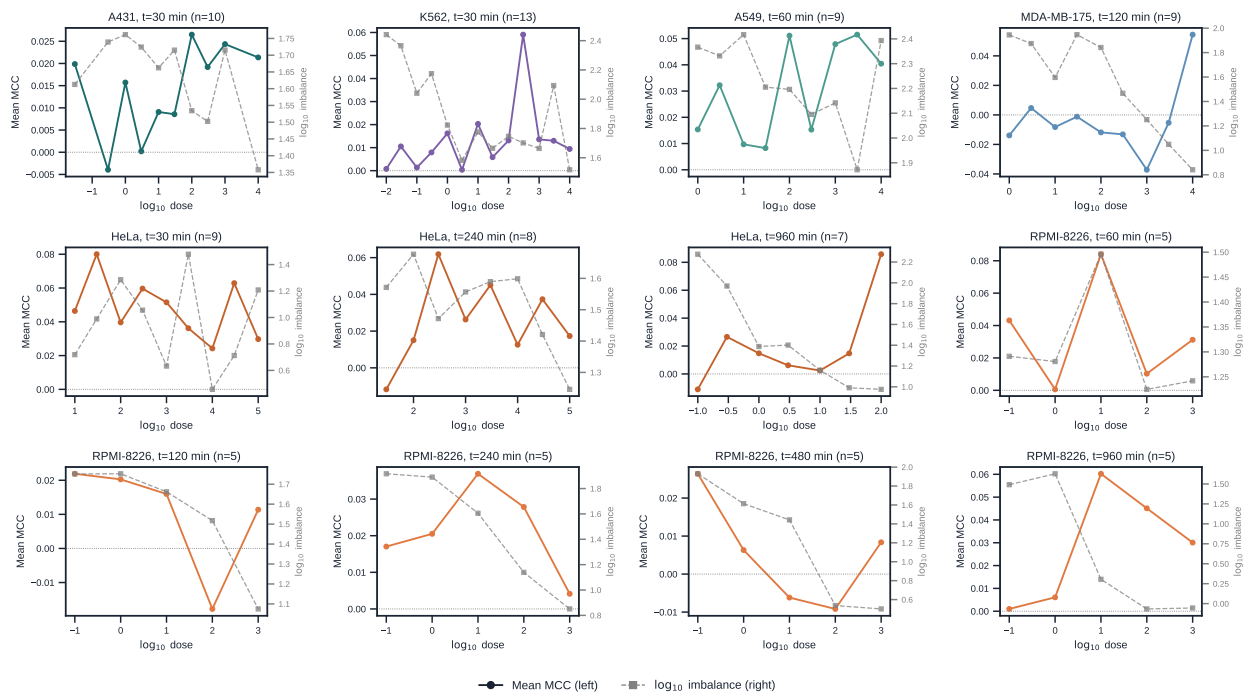

Figure S3. Configurations with fixed cell-line and treatment duration. Colored lines: mean test-set MCC across the six models (left-axis). grey dashed: $log10$ imbalance ratio (right axis).

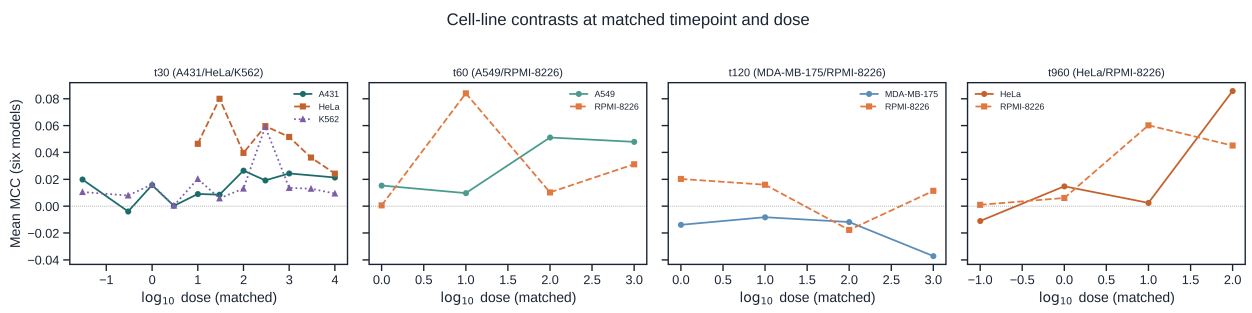

Figure S4. Mean test-set MCC versus matched dose, by cell line, at four timepoints with ≥ 2 cell lines sharing doses. Line styles and markers distinguish cell lines within each panel

= 1, whereas A549 is higher at doses 2 and 3. A similar crossover is observed at 960 min, where RPMI8226 performs better at intermediate doses but HeLa achieves the highest MCC at the largest matched dose. At 120 min, RPMI8226 generally outperforms MDA-MB-175, although both exhibit weak or negative MCC at some doses. Together, these matched comparisons show that cell-line identity affects predictive performance, but the direction and magnitude of this effect depend on dose and timepoint rather than following a fixed cell-line ranking. Thus, even after matching nominal dose and treatment duration, model performance remains strongly context dependent.

Interpretation across controlled analyses

Taken together, the controlled analyses indicate that the observed imbalance ratio captures the frequency of regulated PTM events but does not fully characterize their predictive separability. Dose, treatment duration, and cellular background alter the underlying PTM-response landscape in different ways, while model performance additionally depends on whether those biological differences a re r epresented i n t he available protein and drug features and can be exploited by the model architecture. Thus, predictive difficulty in DrugPTM-Bench should be viewed as the joint consequence of class imbalance, biological heterogeneity, feature representation, model inductive bias, and experimental/label uncertainty rather than as a function of any single experimental variable.

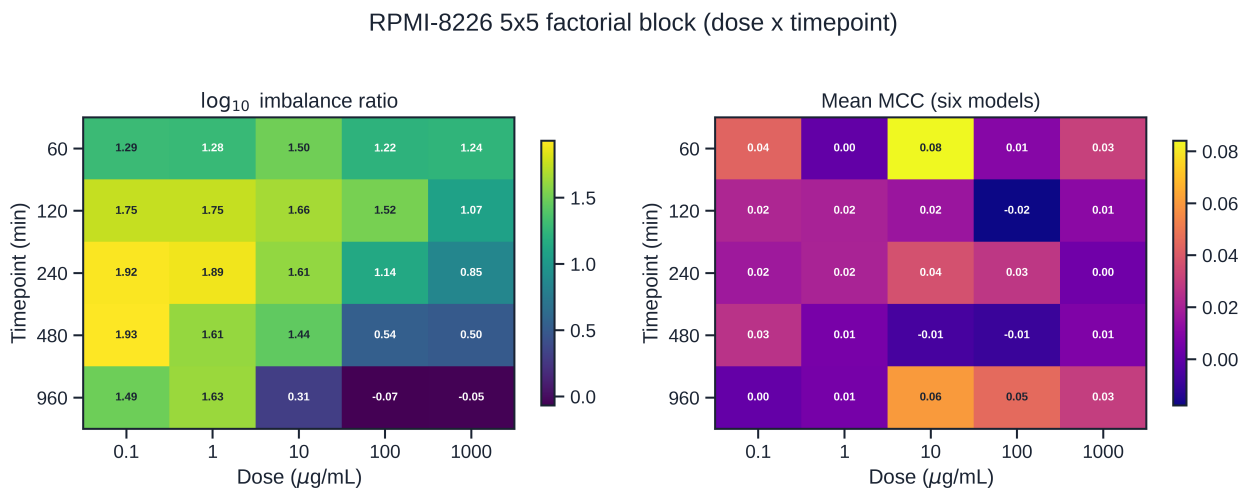

Figure S5. RPMI8226 5×5 factorial block. Left: $log10$ imbalance ratio by dose and timepoint. Right: mean test-set MCC across the six models.

Evaluation Composition and Uncertainty Estimates

This section addresses two related questions: which records were entered into each reported evaluation, and how precisely the resulting metrics are estimated.

Table S4 distinguishes the full set of labelled measurements available for each representative configuration from the protein-disjoint held-out partition on which the models were actually scored. The held-out partition retains between 6.4% and 6.6% of the configuration rows. Minority-class support in the evaluated partition is substantially smaller than the configuration-level counts. In the Low setting the Up class is represented by two sites, both of which reside on a single protein (UniProt P42166).

Tables S5 and S6 report 95% confidence intervals obtained by resampling unique test-set proteins with replacement (1,500 resamples, seed 42), with all sites measured on a given protein resampled together as a block. In the Low setting, for example, 1,636 test rows originate from only 212 proteins.

Two consequences are worth noting. First, because both Up-class sites in the Low setting lie on the same protein, AUPRC for that class is undefined in 37.1% of resamples, closely matching the $(1 - 1/212)^{212} \approx 1/e$ probability that the protein is omitted. The corresponding intervals are degenerate and are reported for completeness only. Second, for the ranking-based metrics the no-signal null is 1.0 rather than 0, and intervals whose lower bound falls below 1.0 are not distinguishable from random ranking.

Table S4. Composition of the held-out test set used for benchmark evaluation. The test set consists of a protein-disjoint partition of each experimental configuration. Minority-class support is substantially smaller than in the full dataset, with the Up class represented by only two sites in the Low setting.

Setting	Cell line	Dose (μg)	Time (min)	Rows	Prot.	Neut.	Up	Down
Severe	K562	0.1	30	3,218	269	3,189	17	12
Medium	A431	10	30	11,124	366	10,950	63	111
Low	RPMI8226	100	60	1,636	212	1,544	2	90

Table S5. Protein-clustered bootstrap 95% confidence intervals for MCC and AUPRC across the three representative settings. Intervals were obtained by resampling unique test-set proteins with replacement (1,500 resamples, seed 42), so that all PTM sites measured on the same protein travel together as a block. Values are reported as point estimate (lower bound, upper bound). n_{test} and n_{prot} give the number of test rows and unique test proteins actually entered into each evaluation. S_{Up} and S_{Down} give the corresponding minority-class support counts.

Setting	Model	n_{test}	n_{prot}	S_{Up}	S_{Down}	MCC	AUPRC (Up)	AUPRC (Down)
Severe	DT	3,218	269	17	12	0.009 (−0.014, 0.054)	0.005 (0.002, 0.009)	0.008 (0.002, 0.038)
Severe	DT-ensemble	3,218	269	17	12	0.005 (−0.021, 0.032)	0.006 (0.003, 0.017)	0.006 (0.003, 0.015)
Severe	RF	3,218	269	17	12	0.000 (0.000, 0.000)	0.009 (0.003, 0.021)	0.018 (0.002, 0.055)
Severe	RF-ensemble	3,218	269	17	12	−0.009 (−0.032, 0.018)	0.014 (0.002, 0.066)	0.010 (0.002, 0.050)
Severe	Dual Encoder	3,218	269	17	12	0.013 (−0.013, 0.064)	0.039 (0.002, 0.210)	0.004 (0.002, 0.015)
Severe	Dual Encoder Focal	3,218	269	17	12	−0.009 (−0.013, −0.006)	0.008 (0.002, 0.037)	0.004 (0.002, 0.009)
Medium	DT	4,273	328	54	37	0.000 (−0.021, 0.026)	0.014 (0.009, 0.026)	0.009 (0.006, 0.012)
Medium	DT-ensemble	4,273	328	54	37	0.010 (−0.012, 0.035)	0.016 (0.011, 0.026)	0.011 (0.006, 0.019)
Medium	RF	4,273	328	54	37	0.000 (0.000, 0.000)	0.013 (0.008, 0.021)	0.010 (0.006, 0.015)
Medium	RF-ensemble	4,273	328	54	37	−0.002 (−0.030, 0.025)	0.014 (0.009, 0.024)	0.009 (0.006, 0.016)
Medium	Dual Encoder	11,124	366	63	111	0.024 (−0.011, 0.047)	0.010 (0.006, 0.017)	0.013 (0.007, 0.018)
Medium	Dual Encoder Focal	11,124	366	63	111	0.022 (0.007, 0.037)	0.007 (0.004, 0.013)	0.011 (0.007, 0.015)
Low	DT	1,636	212	2	90	0.008 (−0.042, 0.063)	0.001 (0.001, 0.004)	0.057 (0.029, 0.090)
Low	DT-ensemble	1,636	212	2	90	−0.021 (−0.060, 0.024)	0.003 (0.002, 0.009)	0.049 (0.025, 0.088)
Low	RF	1,636	212	2	90	0.000 (0.000, 0.000)	0.003 (0.002, 0.008)	0.061 (0.030, 0.106)
Low	RF-ensemble	1,636	212	2	90	0.016 (−0.030, 0.080)	0.003 (0.002, 0.010)	0.060 (0.030, 0.100)
Low	Dual Encoder	1,636	212	2	90	−0.004 (−0.054, 0.066)	0.002 (0.002, 0.007)	0.085 (0.042, 0.166)
Low	Dual Encoder Focal	1,636	212	2	90	0.063 (0.003, 0.143)	0.001 (0.001, 0.004)	0.089 (0.040, 0.201)

Table S6. Protein-clustered bootstrap 95% confidence intervals for the ranking-based metrics. Prevalence-relative AUPRC is AUPRC divided by class prevalence. EF@1% is the top-1% enrichment factor. For both quantities the no-signal null is 1.0 rather than 0, so an interval whose lower bound falls below 1.0 is not distinguishable from random ranking. Resampling protocol is identical to Table S5. In the Low setting the Up class has a support of 2, both sites residing on a single protein (UniProt P42166). The Up-class interval is therefore degenerate and is reported for completeness only.

		Prevalence-relative AUPRC		EF@1%	
Setting	Model	Up	Down	Up	Down
Severe	DT	1.00 (1.00, 1.00)	2.09 (1.00, 10.86)	0.00 (0.00, 0.00)	8.38 (0.00, 23.11)
Severe	DT-ensemble	1.22 (0.85, 2.24)	1.63 (1.09, 4.41)	0.00 (0.00, 0.00)	0.00 (0.00, 0.00)
Severe	RF	1.69 (1.16, 3.25)	4.93 (0.86, 22.23)	0.00 (0.00, 0.00)	8.38 (0.00, 30.17)
Severe	RF-ensemble	2.66 (0.94, 10.24)	2.73 (0.91, 17.24)	5.92 (0.00, 24.84)	8.38 (0.00, 29.83)
Severe	Dual Encoder	7.40 (0.72, 40.26)	1.13 (0.72, 5.24)	11.83 (0.00, 30.61)	0.00 (0.00, 12.10)
Severe	Dual Encoder Focal	1.60 (0.68, 5.02)	1.13 (0.81, 2.80)	0.00 (0.00, 0.00)	0.00 (0.00, 0.00)
Medium	DT	1.10 (0.99, 2.29)	0.99 (0.99, 1.14)	1.84 (0.00, 7.37)	0.00 (0.00, 0.00)
Medium	DT-ensemble	1.27 (1.08, 1.82)	1.24 (0.90, 1.97)	0.00 (0.00, 0.00)	0.00 (0.00, 0.00)
Medium	RF	1.05 (0.93, 1.31)	1.12 (0.95, 1.58)	0.00 (0.00, 0.00)	0.00 (0.00, 0.00)
Medium	RF-ensemble	1.13 (0.93, 1.59)	1.09 (0.88, 1.61)	0.00 (0.00, 0.00)	0.00 (0.00, 0.00)
Medium	Dual Encoder	1.71 (1.30, 3.76)	1.26 (0.91, 1.71)	1.59 (0.00, 7.38)	1.81 (0.00, 2.49)
Medium	Dual Encoder Focal	1.22 (0.91, 2.91)	1.15 (0.88, 1.46)	1.59 (0.00, 6.89)	1.81 (0.00, 3.61)
Low	DT	1.00 (1.00, 1.00)	1.03 (0.99, 1.25)	0.00 (0.00, 0.00)	1.14 (0.00, 4.50)
Low	DT-ensemble	2.18 (1.70, 2.76)	0.90 (0.74, 1.27)	0.00 (0.00, 0.00)	0.00 (0.00, 0.00)
Low	RF	2.12 (1.70, 2.53)	1.10 (0.88, 1.72)	0.00 (0.00, 0.00)	0.00 (0.00, 1.72)
Low	RF-ensemble	2.16 (1.77, 2.72)	1.09 (0.85, 1.71)	0.00 (0.00, 0.00)	2.27 (0.00, 4.38)
Low	Dual Encoder	1.57 (1.29, 1.94)	1.54 (1.05, 3.12)	0.00 (0.00, 0.00)	3.41 (0.00, 9.78)
Low	Dual Encoder Focal	0.89 (0.84, 0.96)	1.62 (1.02, 3.91)	0.00 (0.00, 0.00)	2.27 (0.00, 7.86)

References

1. Pedro Beltrao, Véronique Albanèse, Lillian R Kenner, Danielle L Swaney, Alma Burlingame, Judit Villén, Wendell A Lim, James S Fraser, Judith Frydman, and Nevan J Krogan. Systematic functional prioritization of protein posttranslational modifications. *Cell*, 150(2):413–425, 2012.
2. Eugene F Douglass, Robert J Allaway, Bence Szalai, Wenyu Wang, Tingzhong Tian, Adrià Fernández-Torras, Ron Realubit, Charles Karan, Shuyu Zheng, Alberto Pessia, et al. A community challenge for a pancancer drug mechanism of action inference from perturbational profile data. *Cell Reports Medicine*, 3(1), 2022.
3. Feng Ge, Chuan-Le Xiao, Li-Jun Bi, Sheng-Ce Tao, Sheng Xiong, Xin-Feng Yin, Li-Ping Li, Chun-Hua Lu, Hai-Tao Jia, and Qing-Yu He. Quantitative phosphoproteomics of proteasome inhibition in multiple myeloma cells. *PLoS One*, 5(9):e13095, 2010.
4. Peter V Hornbeck, Jon M Kornhauser, Vaughan Latham, Beth Murray, Vidhisha Nandhikonda, Alex Nord, Elżbieta Skrzypek, Travis Wheeler, Bin Zhang, and Florian Gnad. 15 years of phosphositeplus®: integrating post-translationally modified sites, disease variants and isoforms. *Nucleic acids research*, 47(D1):D433–D441, 2019.
5. Gwanghoon Jang, Sungjoon Park, Sanghoon Lee, Sunkyu Kim, Sejeong Park, and Jaewoo Kang. Predicting mechanism of action of novel compounds using compound structure and transcriptomic signature coembedding. *Bioinformatics*, 37(Supplement_1):i376–i382, 2021.
6. Likun Jiang, Susu Qu, Zhengqiu Yu, Jianmin Wang, and Xiangrong Liu. Moasl: Predicting drug mechanism of actions through similarity learning with transcriptomic signature. *Computers in Biology and Medicine*, 169:107853, 2024.
7. Jared L Johnson, Tomer M Yaron, Emily M Huntsman, Alexander Kerelsky, Junho Song, Amit Regev, Ting-Yu Lin, Katarina Liberatore, Daniel M Cizin, Benjamin M Cohen, et al. An atlas of substrate specificities for the human serine/threonine kinome. *Nature*, 613(7945):759–766, 2023.
8. Patrick H Kennedy, Amin Alborzian Deh Sheikh, Matthew Balakar, Alexander C Jones, Meagan E Olive, Mudra Hegde, Maria I Matias, Natan Pirete, Rajan Burt, Jonathan Levy, et al. Post-translational modification-centric base editor screens to assess phosphorylation site functionality in high throughput. *Nature methods*, 21(6):1033–1043, 2024.
9. Dylan C Mitchell, Miljan Kuljanin, Jiaming Li, Jonathan G Van Vranken, Nathan Bulloch, Devin K Schweppe, Edward L Huttlin, and Steven P Gygi. A proteome-wide atlas of drug mechanism of action. *Nature Biotechnology*, 41(6):845–857, 2023.
10. David Ochoa, Andrew F Jarnuczak, Cristina Viéitez, Maja Gehre, Margaret Soucheray, André Mateus, Askar A Kleefeldt, Anthony Hill, Luz Garcia-Alonso, Frank Stein, et al. The functional landscape of the human phosphoproteome. *Nature biotechnology*, 38(3):365–373, 2020.
11. Aravind Subramanian, Rajiv Narayan, Steven M Corsello, David D Peck, Ted E Natoli, Xiaodong Lu, Joshua Gould, John F Davis, Andrew A Tubelli, Jacob K Asiedu, et al. A next generation connectivity map: L1000 platform and the first 1,000,000 profiles. *Cell*, 171(6):1437–1452, 2017.
12. Jing Tang, Agnieszka Szwajda, Sushil Shakyawar, Tao Xu, Petteri Hintsanen, Krister Wennerberg, and Tero Aittokallio. Making sense of large-scale kinase inhibitor bioactivity data sets: a comparative and integrative analysis. *Journal of chemical information and modeling*, 54(3):735–743, 2014.
13. Jana Zecha, Florian P Bayer, Svenja Wiechmann, Julia Woortman, Nicola Berner, Julian Müller, Annika Schneider, Karl Kramer, Mar Abril-Gil, Thomas Hopf, et al. Decrypting drug actions and protein modifications by dose- and time-resolved proteomics. *Science*, 380(6640):93–101, 2023.
